# Gene Expression Noise in Spatial Patterning: *hunchback* Promoter Structure Affects Noise Amplitude and Distribution in *Drosophila* Segmentation

**DOI:** 10.1371/journal.pcbi.1001069

**Published:** 2011-02-03

**Authors:** David M. Holloway, Francisco J. P. Lopes, Luciano da Fontoura Costa, Bruno A. N. Travençolo, Nina Golyandina, Konstantin Usevich, Alexander V. Spirov

**Affiliations:** 1 Mathematics Department, British Columbia Institute of Technology, Burnaby, British Columbia, Canada; 2 Biology Department, University of Victoria, Victoria, British Columbia, Canada; 3 Instituto de Biofisica, Universidade Federal do Rio de Janeiro, Rio de Janeiro, Brazil; 4 Instituto de Fisica de Sao Carlos, Universidade de Sao Paulo, Sao Carlos, Sao Paulo, Brazil; 5 Faculty of Computing, Federal University of Uberlândia, Uberlândia, Brazil; 6 Mathematics and Mechanics Faculty, St. Petersburg State University, St. Petersburg, Russia; 7 Computer Science and Center of Excellence in Wireless and Information Technology, Stony Brook University, Stony Brook, New York, United States of America; Washington University in Saint Louis, United States of America

## Abstract

Positional information in developing embryos is specified by spatial gradients of transcriptional regulators. One of the classic systems for studying this is the activation of the *hunchback* (*hb*) gene in early fruit fly (*Drosophila*) segmentation by the maternally-derived gradient of the Bicoid (Bcd) protein. Gene regulation is subject to intrinsic noise which can produce variable expression. This variability must be constrained in the highly reproducible and coordinated events of development. We identify means by which noise is controlled during gene expression by characterizing the dependence of *hb* mRNA and protein output noise on *hb* promoter structure and transcriptional dynamics. We use a stochastic model of the *hb* promoter in which the number and strength of Bcd and Hb (self-regulatory) binding sites can be varied. Model parameters are fit to data from WT embryos, the self-regulation mutant *hb* ^14F^, and lacZ reporter constructs using different portions of the *hb* promoter. We have corroborated model noise predictions experimentally. The results indicate that WT (self-regulatory) Hb output noise is predominantly dependent on the transcription and translation dynamics of its own expression, rather than on Bcd fluctuations. The constructs and mutant, which lack self-regulation, indicate that the multiple Bcd binding sites in the *hb* promoter (and their strengths) also play a role in buffering noise. The model is robust to the variation in Bcd binding site number across a number of fly species. This study identifies particular ways in which promoter structure and regulatory dynamics reduce *hb* output noise. Insofar as many of these are common features of genes (e.g. multiple regulatory sites, cooperativity, self-feedback), the current results contribute to the general understanding of the reproducibility and determinacy of spatial patterning in early development.

## Author Summary

Noise is an intrinsic part of biochemical systems such as gene regulation networks. Noisy gene expression has been well documented in populations of single cells, and is likely a key mechanism in evolutionary change. But in developing embryos, cells within a tissue must overcome such variability in order to provide the uniformity required to coordinate multiple events. Reproducibility and determinacy of the spatial protein patterns preceding tissue differentiation is a critical aspect of development. In this study, we use anterior-posterior (AP) segmentation in the fruit fly (*Drosophila*) to understand how gene regulation dynamics control noise. One of the earliest AP patterning events is the anterior activation of the *hunchback* (*hb*) gene by the maternally-derived Bicoid (Bcd) protein gradient. This interaction has been very well characterized, providing the tools for us to develop a stochastic model of *hb* gene regulation to make predictions about expression noise, and to corroborate these experimentally. For *hb*, we show that self-regulation is a critical part of controlling noise, and the multiple Bcd binding sites in the *hb* promoter also enhance pattern reproducibility. To the degree that such features are shared by other genes, these noise-reducing mechanisms may be common to many pattern forming events.

## Introduction

**Figure 1:**
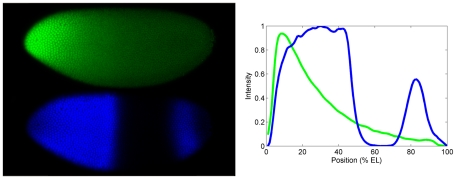
Concentration profiles of the morphogenetic proteins Bicoid (Bcd) and Hunchback (Hb). (A) A *Drosophila* embryo fluorescently immunostained for Bcd (green) and Hb (blue), about 30 minutes into nuclear cleavage cycle 14. Anterior left, dorsal top. Nuclei at the surface of the precellular, syncytial blastoderm are visible. (B) Fluorescence intensity against anterior-posterior (AP) position (in percent egg length (%EL), colours as in A, showing the exponential Bcd gradient and the step-like Hb pattern. From [30].

One of the fundamental questions in biology is how embryos develop reproducibly, and it has many aspects. Here, we focus on the reproducibility of the spatial gene expression patterns which determine the body plan. At a broad level, one can ask what the degree of variability is in a population of embryos - the degree to which parameters controlling developmental patterning can vary before major disruptions occur. In recent years, a number of studies have made quantitative comparisons of developmental patterns between embryos in the fruit fly, *Drosophila melanogaster*, aided by its very well characterized molecular biology. For instance, there has been a recent focus on spatial precision of the maternally-derived Bicoid protein (Bcd; Figure 1, green), which forms an anterior-posterior (AP) concentration gradient [1]–[5]. Bcd is a transcriptional regulator of downstream segmentation genes, and has been studied as a classic example of a positional information gradient, in which alterations in the gradient shift downstream patterns in a concentration-dependent manner [6]–[10]. It has been shown, though, that Bcd has lower spatial precision than its downstream targets: the gap gene *hunchback* (*hb*; Figure 1, blue) has a mid-embryo domain boundary at a position some 2 to 7 times less variable than the corresponding Bcd concentration threshold [1], [4], [5], [11], [12]; and the pair-rule gene *even-skipped* starts out with Bcd-like precision but achieves *hb*-like precision as its pattern develops [3]. In addition, microfluidic temperature experiments have shown robust downstream patterning following extreme disruption of the Bcd gradient [13], [14]; and even with experimentally flattened Bcd gradients, embryos form gap gene patterns in the correct order [15]. All of this suggests that the initial maternal positional information is modified during development in order for expression patterns to achieve necessary levels of precision.

In addition to the precision between embryos, spatial patterns within individual embryos are well-determined, with low cell-to-cell variability (or nucleus-to-nucleus, for the precellular *Drosophila* blastoderm), despite numerous sources of noise. These include: the state of the DNA; mRNA and protein production; intra- and inter-cellular compartmentalization; and cellular movements and ordering. Error control is likely to occur at each these to limit noise and allow development to proceed. Previous studies have investigated aspects of this, for instance, at the DNA level [16] and overall tissue level [17], [18]). In this paper, we focus on the noise which can arise in mRNA and protein production, due to the inherently random nature of reactions at low copy number. This builds on a now extensive literature of gene expression noise in single celled organisms (e.g. [19]–[24]). But by studying *hb* patterning - the initial conversion of the smoothly decreasing Bcd gradient into a sharp and precise zygotic pattern segmenting the body (Figure 1) - we focus on spatial noise: what are the dynamics of noise generation in *hb* patterning (including, in contrast to single cells, randomness in transport between nuclei); and how is noise controlled within the constraints of these dynamics, producing the nucleus-to-nucleus noise levels observed for *hb*? We show that some degree of Hb between-embryo positional variability can arise purely from randomness in transcription and translation. But the larger issue is that gene expression has a strong potential for amplifying the microscopic randomness of low copy number into indeterminate macroscopic patterns within an embryo (i.e. with indistinct or missing boundaries). In the present work, we investigate what dynamic features and parameter ranges are necessary for *hb* expression to overcome this, in order to form determinate pattern.

A broad distinction can be made between gene expression noise that is external, due to fluctuations in upstream regulator concentrations or global parameters (e.g. rate constants), and internal, due to the random nature of reactions (e.g. how many molecules per unit time are created or destroyed) and transport (how many molecules arrive in or leave a unit volume in a given time). Even in the absence of external sources (i.e. with fixed, non-fluctuating inputs) internal sources will cause fluctuating output. The amplification of external noise can potentially be significant in hierarchical signalling, such as in *Drosophila* segmentation; but data [4],[25] indicate that Bcd delivers a relatively non-noisy signal to nuclei (discussed further below), which indicates that much of the observed between-nucleus noise in Hb is generated internally, in the process of mRNA and protein production.

**Figure 2:**
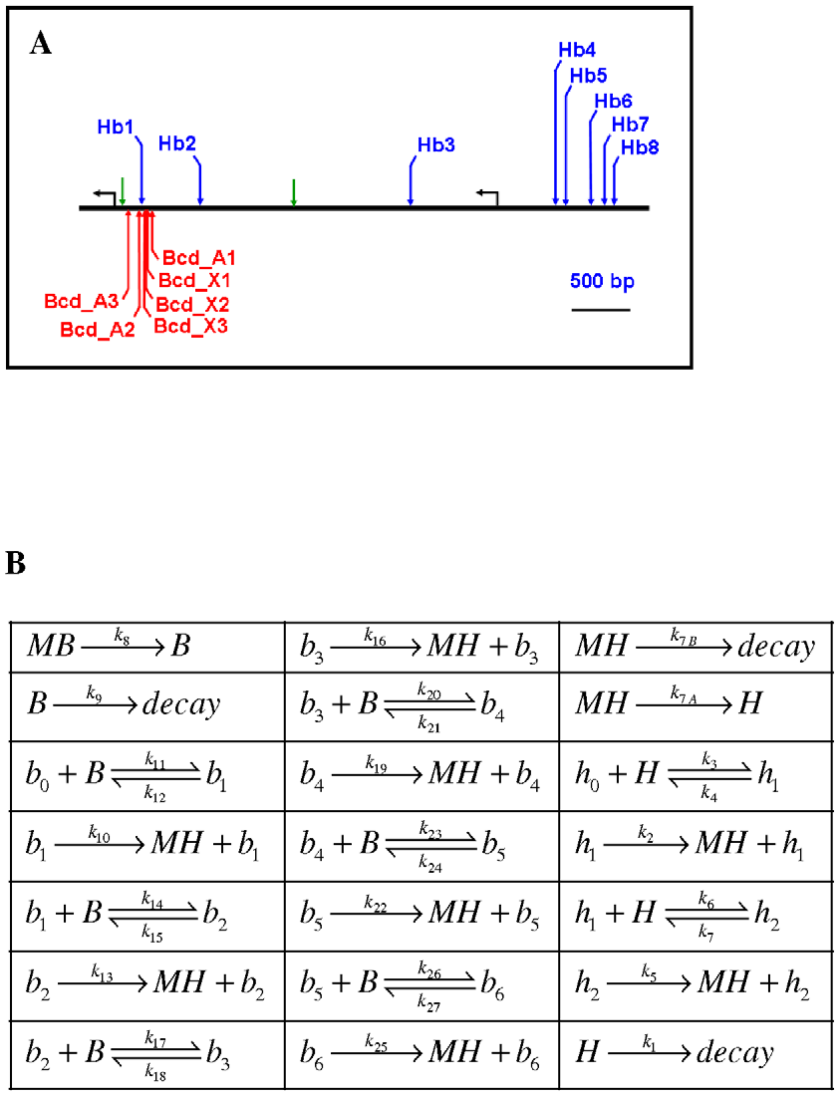
Model of the *hb* promoter. (A) Schematic diagram of a 4776 bp fragment from the *hb* regulatory region determined by in vitro footprinting [31], [32], adapted from [[30], Figure S4]. The Bcd sites (red; A are strongly binding, X are weaker) and first two Hb sites can drive relatively sharp anterior expression in lacZ constructs in a WT background (e.g. Figure 7D). This region (between the green arrows) has been extensively studied as the core of the proximal promoter and is the basis of our model. (B) The reaction network based on these core binding sites, involving binding, unbinding, transcription, translation and decay. *B* = Bcd protein; *H* = Hb protein; *MB* and *MH* are their mRNAs, respectively. *bcd* mRNA is translated at the anterior pole. Bcd and Hb proteins diffuse. *b*_0–6_ are the number of Bcds bound to the *hb* promoter; *h*_0–2_ are the number of Hbs bound to the *hb* promoter. The subscript refers to the binding order based on strength. Values of the rate constants (*k*'s) are constrained by experimental data and are given in Tables S1, S2, S3.

We directly model the noise production in *hb* regulation using a chemical master equation approach [26]–[28]. This treats each reaction and transport event with a probability of occurrence per unit time. At the low copy number of many of the species involved in transcriptional regulation, stochastic dynamics predominate, necessitating such a solution method; dynamics generally become more deterministic for copy numbers in the hundreds and above [29]. Several of us were involved in a previous project developing a detailed model of anterior *hb* expression [30]. This was based on experimental mapping of the *hb* promoter [31], [32], and simulated regulation in a core region of the proximal promoter responsible for anterior zygotic *hb* expression (green arrows, Figure 2A). The model included binding/unbinding at 6 Bcd sites (red, Figure 2A) and 2 Hb self-regulatory sites (blue, Figure 2A); Hb production and diffusion; and Bcd translation (at the anterior pole) and diffusion. The model, solved at the deterministic level, successfully predicted Hb boundary position and sharpness for wild-type (WT) and *bcd* and *hb* mutants; and showed that sharpness depends on bistable dynamics due to *hb* self-regulation. Following validation against these macroscopic features, we are now using the model to investigate noise generation in the *hb* expression dynamics. Figure 2B shows the current version of the model; reactions have been added to explicitly model mRNA synthesis. By simulating regulation at this level of detail we can determine the relative noise contributions of, for instance: binding site number and strength; binding cooperativity; self-regulation; and protein diffusion. Noise is uniquely generated by each of these aspects, and determinate pattern formation depends on their associated parameters (e.g. binding, diffusion, production and decay rates) being within controlled ranges, as well as on the types of dynamics (e.g. binding cooperativity, self-regulation). For the *hb* promoter, there are a number of experimental tools which allow us to distinguish these contributions, including the *hb* ^14F^ mutant [33], whose Hb protein does not bind DNA; and a series of lacZ reporter constructs driven by fragments of the *hb* promoter [34]. Data from these embryos, as well as WT, place constraints on the model parameters, allowing us to deduce their relative contributions to the generation and control of *hb* output noise.

Tkacik et al. [35] recently studied the effects of input (Bcd) noise on Hb output. This approach assumed that Hb output exclusively depends on Bcd, in a Hill-type manner. For some cases (*hb* ^14F^, lacZ constructs), such complete Bcd dependence may apply. For WT, however, Hb self-regulation is significant, and greatly influences the final expression pattern [1], [30]. Very recently, Okabe-Oho et al. [36] published results on a stochastic model of Hb production, following our previous model [30]. They modelled binding of the *hb* promoter, but only considered 4 possible bound-states (including that the 6 Bcds or the 2 Hbs bind simultaneously as groups). Using all of the bound states (all the *b*'s and *h*'s in Figure 2B), has enabled us to build up the binding and production constants from lacZ construct data with from 1 to 6 Bcd binding sites of different strengths, and from 0 to 2 Hb sites (together with WT and *hb* ^14F^ data). This has revealed cooperativity and binding strength effects which could not be addressed in the 4-state system. [35] and [36] focused on input noise due to diffusion of regulators to the promoter. [36] reported that amplification of this type of noise can depend strongly on Hb diffusivity. We see a similar sensitivity to Hb diffusivity, but the reproducibility of Hb boundary sharpness between embryos suggests that diffusivity is highly constrained (i.e. not a variable parameter); and the steep boundary indicates a slow diffusivity, i.e. that it is not optimized for noise reduction. Detection of regulator movement within nuclei is beyond current techniques. But measurements of Bcd concentrations at whole nuclei resolution [4] indicate that nucleus-nucleus Bcd fluctuations should be relatively low. At this spatial resolution (our data is processed into ‘energid’ units, of nucleus plus surrounding cytoplasm), we observe that relative noise is higher in *hb* mRNA than in protein, and that Hb self-regulation (comparing WT with the *hb* ^14F^ mutant) decreases relative noise in the protein output. Neither of these effects depend on input noise: the former (the noise difference between mRNA and protein) shows the effects of translation; the latter highlights the effects of self-feedback. Our model predicted these effects from dynamic principles.

The details of the *hb* promoter structure matter for determining expression noise. We predict that binding/unbinding noise dominates in the absence of self-regulation, and increased binding site number and strength serves to reduce noise in these cases. In WT, though, self-feedback produces a bistable mechanism: this was previously shown to be critical in boundary sharpness [30]; the present work shows how this mechanism also promotes expression into a more deterministic, low-noise regime. These results suggest how evolution may have incorporated binding sites and self-feedback mechanisms to produce output determinate enough for robust development.

## Results

### Experimental data determines model parameters

Published data from WT, the *hb^14F^* mutant and the lacZ constructs indicate probable values of the parameters in the model (Figure 2B). We describe below how the data can be used to determine the rates (*k*-values) sequentially, without the need for a global parameter optimization. This involves deterministic (no noise) solution of the model to match macroscopic features of the data (strength of expression, expression boundary position, boundary sharpness, timing). This parameter set is then used in stochastic solutions of the model to make predictions on noise levels and characteristics, which are corroborated against new experimental data (next section, Stochastic Results). The assumptions involved in the parameter fitting are not expected to affect the noise predictions (see Discussion). The main points on parameter fitting are given here, with further details given in Text S1.

#### Concentration sets production rates

Gregor et al. [4] reported a mid-embryo concentration for Bcd-GFP (Green Fluorescent Protein) of approximately 8 nM, about 700 protein molecules per nucleus (nuclear volume around (5µm)^3^). With the exponential form of the Bcd gradient [3], this corresponds to 7000 molecules per nucleus at the anterior pole. Given these current best measurements of absolute protein concentration in the cycle 14 blastoderm, a reasonable first estimate for the Hb maximum would similarly be in the range of 7000 molecules per nucleus. (Using methods stated to be biased low, Zamparo and Perkins [37] estimated at least 820–1300 Hb molecules per nucleus – and roughly equal to the Bcd concentration range found by their techniques.) The Hb maximum sets the overall transcription rates in the model (*k*_2,5,10,13,16,19,22,25_; Table S1). We have measured protein intensity in the *hb* ^14F^ mutant to be 15% of WT [30]; this difference sets the relative values of the Hb-bound transcription rates (*k*_2,5_). For constructs driven by only Bcd sites, Driever et al. [34] qualitatively scaled the decrease of lacZ intensity with decreasing Bcd site number; coupled with quantitative in vitro data [32], these set the relative values of Bcd-bound transcription (*k*_10,13,16,19,22,25_). The trend from *k*_10_ to *k*_25_ is non-linear, suggesting that the multiple Bcd sites have a synergistic effect on overall transcription rate (particularly for more than 3 Bcds bound).

#### Expression boundaries set Bcd binding constants

In the Driever constructs, lacZ boundary positions shift according to the number of Bcd sites and their strength [34]. Earlier DNAse footprinting mapped 3 strong (A) and 3 weak (X) Bcd binding sites in the proximal *hb* promoter [32; see Figure 2A, red]. The Driever constructs are driven by a number of combinations of A and X sites. Starting from the construct with a single A site, matching the posterior lacZ boundary positions for all the constructs sets the Bcd binding constants in the model (*k*_11,14,17,20,23,26_; Table S2; note that the model numbers the order of binding - 1^st^ Bcd, 2^nd^ Bcd, etc. – not the location at which a particular binding occurs). Comparison, e.g. between constructs with 3A vs. 3X sites, allows us to predict relative differences in strong and weak binding strengths. Earlier in vitro work [38]–[40] identified cooperativity in binding up to 3 Bcds, which we incorporated into the *k*_11,14,17_ values. Modelling the positions for constructs (and *hb* ^14F^) with 4 or more Bcd sites indicates a further cooperativity in these additional bindings.

#### Hb regulation sets the timescale

The posterior shift of the Hb boundary (specifically the posterior, mid-embryo boundary) from *hb* ^14F^ to WT sets the binding constants for the 2 Hb sites in the model (Table S3). The Hb boundary position is fairly steady in WT, over a period of about 5 to 40 minutes into nuclear cleavage cycle 14 [30, Figure 2CD]. At the same time, sharpness increases by about 20°, reaching steady values near 30 minutes (sharpening occurs before the later Hb patterning in the posterior and at parasegment 4: it is driven by the proximal promoter, Figure 2A green arrows). This sets the timescale of Hb production and decay: the steady sharpness and maximum depend on the ratio of Hb production to decay, but reaching steady values by 30 minutes constrains the absolute values of these rate constants (faster production and decay reach steady state faster). Simulations were initiated with experimental Hb data from the onset of cycle 14, about 65% of the mature cycle 14 maximum. The Hb protein diffusivity is also constrained by observed sharpness: if diffusivity is too high, self-regulation cannot sharpen the boundary - WT simulations become only marginally sharper than *hb* ^14F^ (experimental data shows a 15° difference); if diffusivity is too low, the boundary becomes sharper than experiment. A Hb diffusivity of 0.3µm^2^/s (equal to the value measured in [41] for Bcd-GFP diffusion in the vicinity of nuclei) best fits the experimental observations. Results from [36] suggested fast Hb diffusion as a means of decreasing noise, but the sharpness and reproducibility of Hb profiles [[30], Figure 1] indicate that diffusivity is tightly constrained, and may not be optimized for noise control.

### Stochastic results

With the parameters thus determined, we modelled WT, *hb* ^14F^ and the lacZ constructs to predict the mRNA and protein noise arising from different aspects of transcription and translation.

#### Wild-type noise

**Figure 3:**
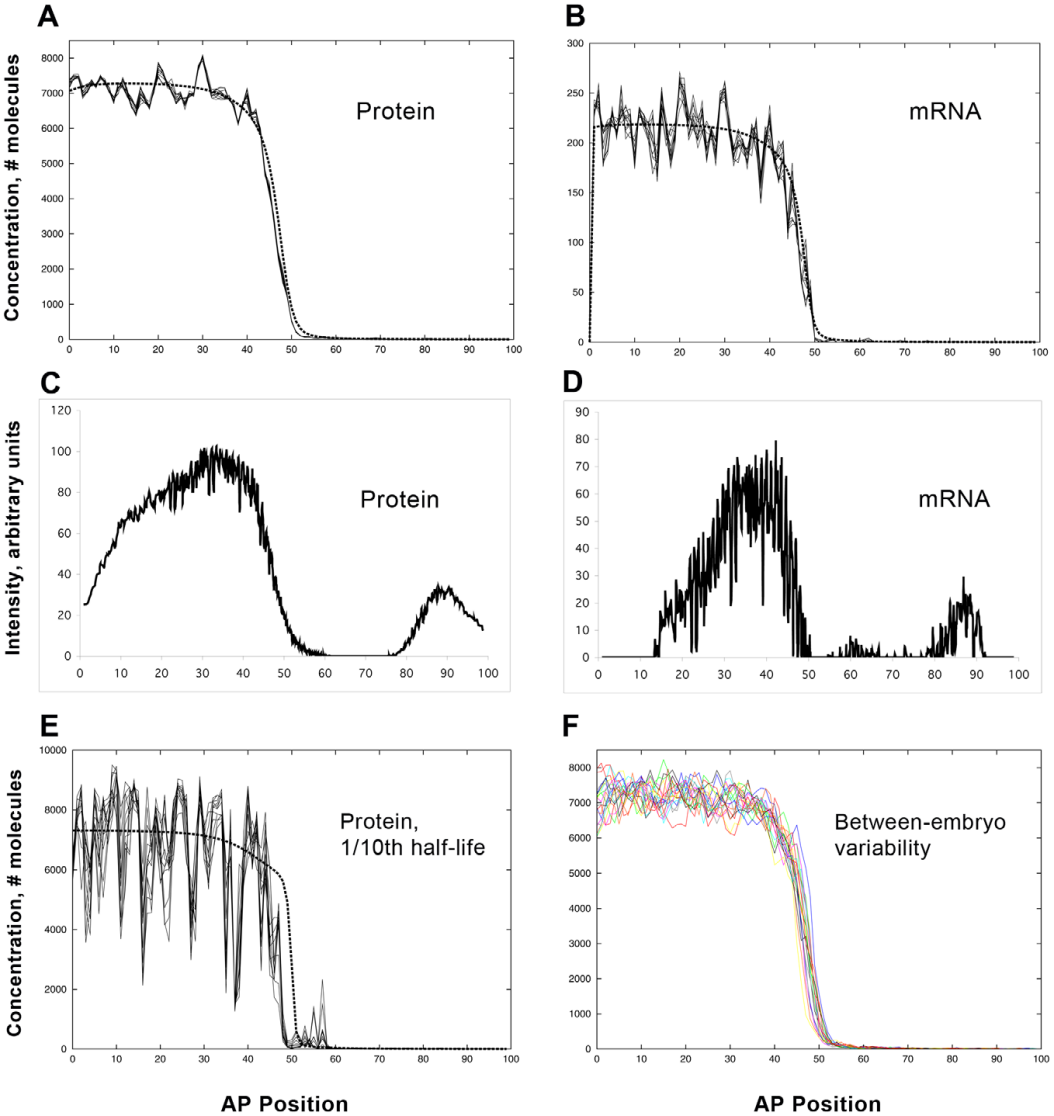
Wild-type (WT) expression noise. (A, B) *hb* protein and mRNA profiles, respectively, from the model (Figure 2B; 6 Bcd sites (3A, 3X) and 2 Hb sites, or 6B2H), with parameters fit by experimental constraints (Tables S1, S2, S3). Deterministic (no noise) results, dashed line. Stochastic results are shown over one minute (29–30 minutes into cycle 14), at 5 second intervals (comparable to the experimental heat-fixation time), to show the slow temporal variability of the noise (same format used in Figures 4, 6, 7). Noise is calculated from the differences between the deterministic and stochastic results at 30 minutes (Table 1). (C, D) Representative data from a single WT embryo, *hb* protein and mRNA, respectively, 30–36 minutes into cycle 14 (same time in Figures 4, 6–8). This shows the characteristic determinate mid-embryo boundary, especially for the protein, which is also produced by the model. mRNA (B, D) has significantly higher noise than protein (see Table 1 for statistics). (E) Too-fast reaction rates are one factor that can cause noise to overwhelm determinate expression. Here, reaction rates (transcription, translation, mRNA and protein decay) have been increased ten-fold from (A, B), producing much higher fluctuation levels (protein noise 25%, mRNA noise 32% - higher than any WT results (Table 1)) and reducing the determinacy of the mid-embryo boundary. (F) Within-embryo noise contribution to between-embryo variability: 19 independent stochastic simulations of WT protein expression, with a standard deviation in boundary position of 1.0%EL (comparable to experimentally observed between-embryo variability).

Figure 3 compares model results and experimental data for WT *hb* mRNA and protein. These simulations do not include Bcd noise: the trends were identical between simulations with or without Bcd noise (at levels indicated by the data). The relative independence of Bcd and *hb* noise is explored more fully below. Here, we highlight the *hb* mRNA and protein differences arising from transcription and translation. Figures 3A (protein) and 3B (mRNA) show *hb* output for a WT simulation; experimental data from a WT embryo is shown in Figures 3C and D (protein and mRNA, respectively). Noise statistics (Table 1) are based on concentration differences (residuals): for simulations, from the difference between the stochastic and deterministic (dashed line) solutions at a particular time-point (*t* = 30 minutes, here); for experiments, from the difference between data and fit trends (see Methods). Noise is calculated as the standard deviation of the relative residuals in the activated region (15–45 percent egg length (%EL)). Since the model does not include experimental sources of error, noise comparisons are in terms of relative trends, not absolute levels. To show the temporal stability of the noise, stochastic results are displayed at 5 second intervals over the final minute of computation.

**Table 1:** Noise levels for WT expression, in time.

Time	Model, mRNA^a^	Model, protein^a^	Experiment, mRNA^b^	Experiment, protein^b^
30mins	11 (1.2) %	5.3 (0.92) %	47 (22) %	5.1 (0.89) %
20mins	13 (1.7) %	6.3 (1.7) %		
10mins	14 (1.4) %	7.2 (1.5) %		

Noise is a standard deviation using relative residuals, calculated by 
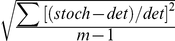
, where *stoch* and *det* are the stochastic and deterministic solutions at each position (energid), respectively; for experimental data, replace *stoch* with background-removed intensity and *det* with the trend found by 2D SSA (see Methods). This measure is calculated for the activated region, 15–45%EL (proximal promoter dependent).

a Average of the noise levels (standard deviation in brackets) for the 19 simulations shown in Figure 3F. mRNA was noisier than protein in every simulation (and p<0.01, for t-test on differences, for each time). The simulation in Figure 3AB has average noise levels (11% for mRNA, 5.4% for protein).

b *n* = 3 embryos. mRNA was noisier than protein in each embryo (and p<0.05 for t-test). The embryo in Figure 3CD has noise of 22% for mRNA and 4.8% for protein. (Limiting analysis to the strongest mRNA expressing region, 30–45%EL, average mRNA noise decreases (to 25%) and becomes less variable (8.0% std. dev.), while protein noise remains stable (mean, 5.7%; std. dev., 1.3%), increasing the significance for their difference.)

Computations generate the characteristic determinate protein boundary seen in the data. For mRNA, the model produces a boundary that is both sharper and noisier than protein, as seen in the data. The model predicts *hb* mRNA should have higher noise than its protein, and this is corroborated in the experimental data (statistical significance, Table 1). Temporally, the simulations suggest a mild decrease in mRNA and protein noise as pattern develops; noise levels for experimental protein data appear steady in the first 30 minutes of cycle 14.

Many factors can affect noise. We found that *hb* self-regulation has a major effect, which is explored in detail in the next section. Other factors identified in preliminary computations were: diffusivity - the faster that Hb protein is transported, the more it smoothes local fluctuations, though at the expense of boundary sharpness (see also [36]); cooperativity - if binding strength increases too much for each sequential Bcd (or Hb) bound, there can be runaway binding events in the posterior half of the embryo, with ‘spikes’ of activation in nuclei which should be ‘off’; reaction rate - the faster that reactions occur, the more the protein concentration displays the high noise conditions (due to low numbers of binding sites) of the promoter. As an example of this, Figure 3E shows a simulation in which mRNA and protein production and decay constants were increased by a factor of 10. Protein timescales are the most critical in this: WT protein expression could be generated with all *hb* mRNA rates increased by a factor of 1000, but Hb pattern was rapidly destroyed if translation and protein decay were moderately sped up (as in Figure 3E). These potential noise sources were largely eliminated by the parameter fitting described above; i.e. matching macroscopic features produced a model parameter set which generated similar noise levels (or determinacy) to that observed in WT – perhaps reflecting the biological selection against parameter values (i.e. rates) which generate noise and threaten pattern.

In between-embryo studies, the drop in positional variability from Bcd to Hb has been noted [1], [5], [12], [42], from mid-embryo standard deviations on the order of 2 to 7%EL for Bcd to about 1.0%EL for the Hb boundary. Not all of the Hb variability may be due to Bcd, however. Figure 3F shows the protein output for 19 independent stochastic WT simulations (all with identical parameters, including identical Bcd). The standard deviation of the boundary position is 1.0%EL for this sample – comparable to values measured between embryos – suggesting that a substantial proportion of between-embryo variability could stem from intrinsic fluctuations in expression dynamics.

#### *hb* self-regulation decreases noise

**Figure 4:**
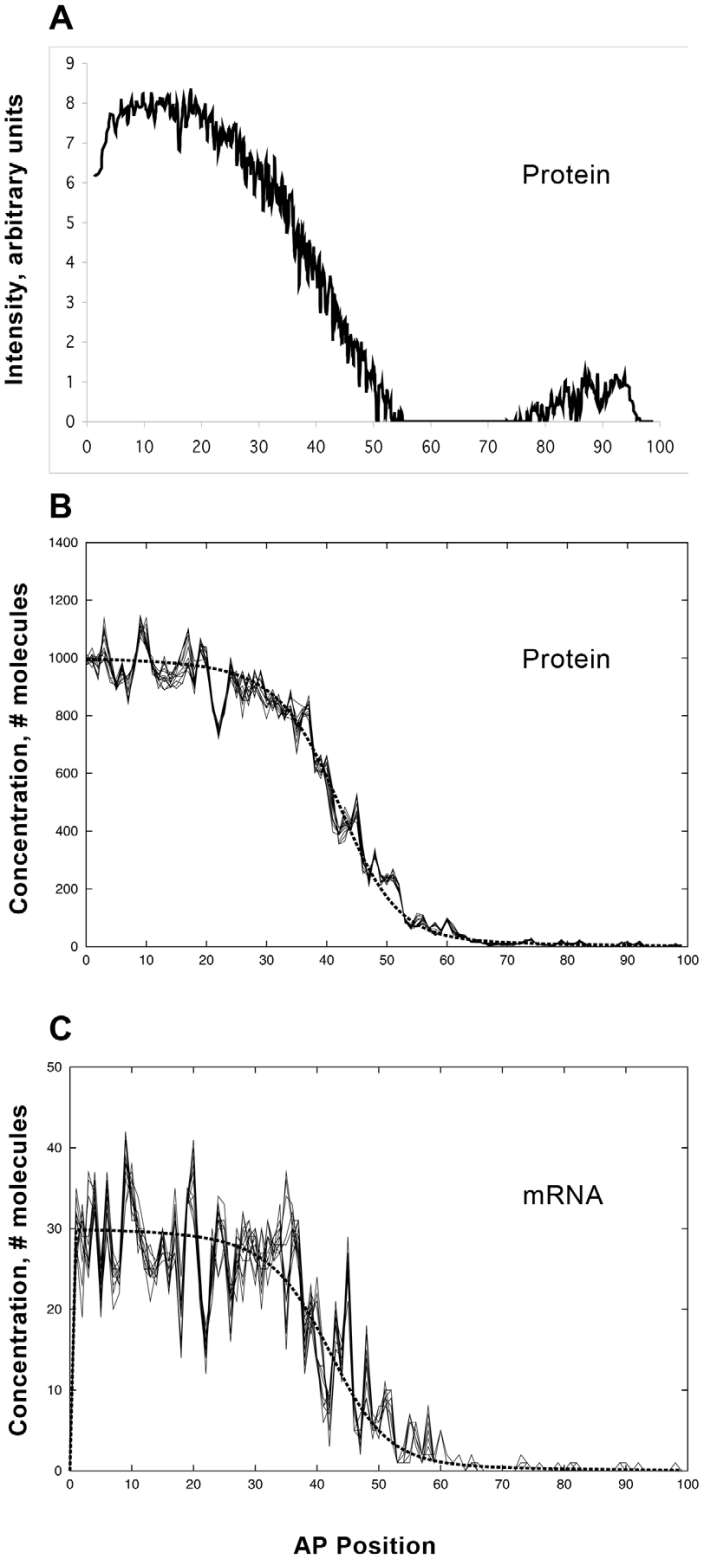
The absence of *hb* self-regulation. (A) Protein expression in a homozygous *hb* ^14F^ mutant embryo (whose Hb protein cannot bind DNA); noise is significantly higher than WT (c.f. Figure 3C). (B) Simulation of *hb^14F^* protein expression: 6 Bcd binding sites and no Hb binding in the promoter (6B0H). Noise is higher than WT. (C) mRNA for the same simulation: noise is higher than protein (B), and higher than for WT mRNA (Figure 3B). See Table 2 for statistics.

*hb* ^14F^ mutants lack self-regulation, and show much lower protein intensity and slope than WT (Figure 4A; Tables S1, S2, and S3). The mutation is also associated with increased noise: simulating *hb* ^14F^ (Figures 4B and C; statistics in Table 2) by not allowing Hb binding in the promoter significantly increases both mRNA and protein noise from WT (c.f. Figures 3A and B; Table 1). (And *hb* ^14F^ model noise is higher for mRNA than protein, as in WT.) The noise increase is corroborated by the data, which show significantly higher protein noise in *hb* ^14F^ (Figure 4A; Table 2) than WT (Figure 3C; Table 1). We would predict that the higher noise in *hb* ^14F^ would combine with its lower slope to produce greater downstream positional errors than WT; therefore that Hb self-regulation plays a dual role of both sharpening the boundary [30] and reducing noise to produce determinate WT pattern.

**Table 2:** Noise levels in the absence of *hb* self-regulation.

Experiment		*hb* ^14F^
		protein^a^
		(Figure 4A)
		8.9 (2.7) %

Model	6B0H^b^	6B0H^b^
	mRNA	protein
	(Figure 4C)	(Figure 4B)
	26 (3.7) %	11 (1.5) %

a *n* = 3 embryos, *hb^14F^* protein noise is higher than WT protein noise (t-test, p<0.05).

b Average of the noise levels (standard deviation in brackets) for 17 simulations with WT Bcd binding but no Hb binding (6B0H), at *t* = 30 mins. mRNA noise was higher than protein in every simulation (and p<0.01, for t-test on differences). The simulation in Figure 4 has average noise levels (29% for mRNA, 11% for protein). Both mRNA and protein noise are higher in 6B0H simulations than in WT simulations (t-test, p<0.01).

The two stages of transcription and translation are also important for clean amplification of the WT self-feedback loop: in preliminary computations, in which only a single generic ‘production’ term was modelled (as in [36]), WT expression was much noisier. Relative fluctuations in the small number of bound sites at the promoter are much higher than in the hundreds of mRNA copies per nucleus (e.g. Figure 3B). Translation from this latter level can help shield the protein from noise at the promoter.

#### Noise characteristics highlight the Bcd-independent aspects of *hb* expression

Probability distributions for species' concentrations are generated by underlying kinetics. Since the master equation approach models each reaction and transport event probabilistically, it generates the unique distributions for each species in a given mechanism. The difference in kinetics between Bcd gradient formation and *hb* expression produces very distinct probability distributions, showing the extent to which Hb noise is produced de novo, independently of Bcd noise.

**Figure 5:**
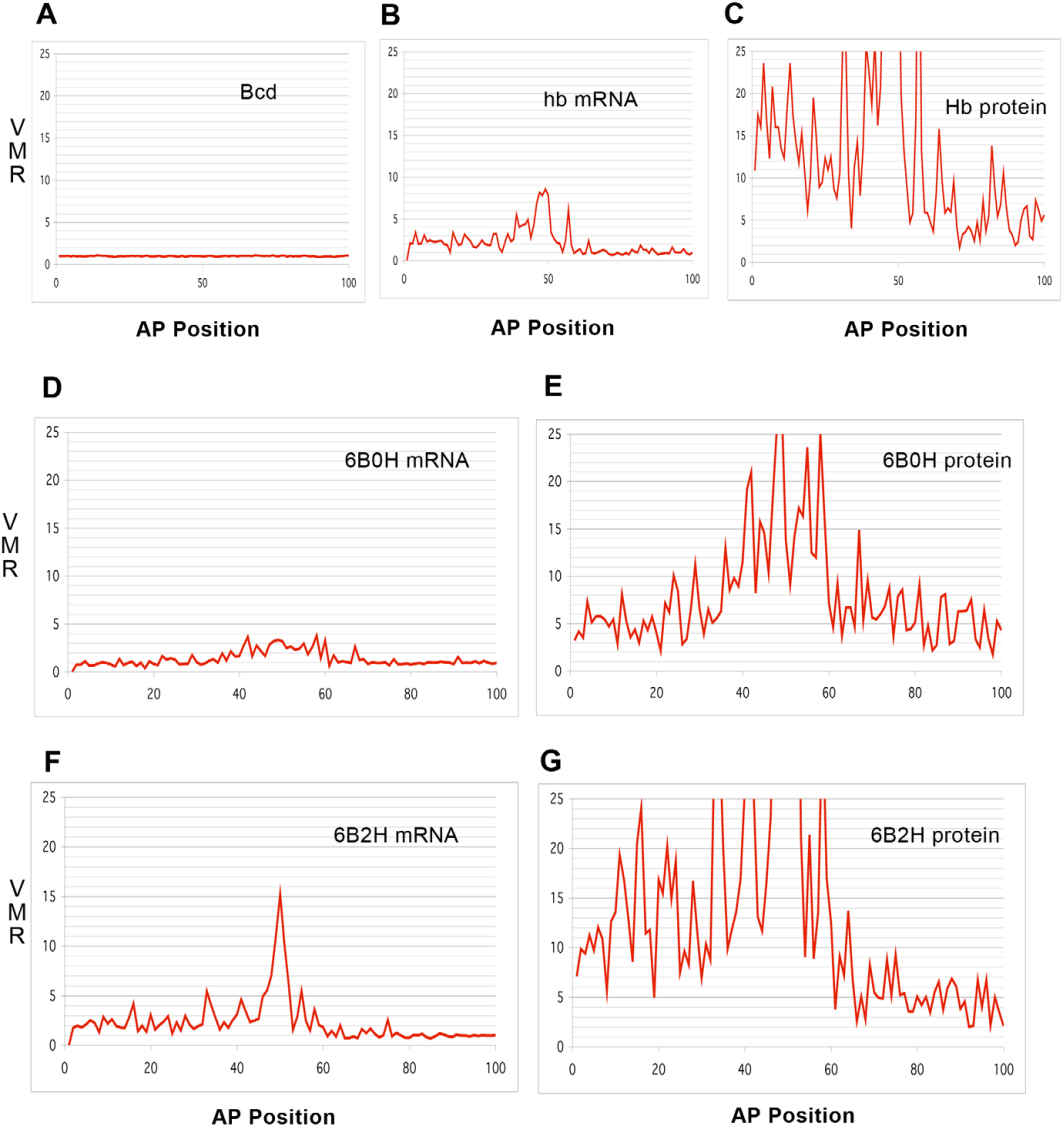
Noise signatures for Bcd, *hb* mRNA and Hb protein. (A, B, C) Variance to mean ratio (VMR) by position, for Bcd (A), *hb* mRNA (B), and Hb protein (C), all from the same computation. Bcd dynamics (synthesis-diffusion-decay) produce a VMR of 1, characteristic of Poisson-distributed noise. *hb* mRNA (B) has a variance 2 to 3 times that for a Poisson process. Translation increases the deviation from Poisson for the protein (C). The non-Poisson character of the mRNA distribution is largely due to Hb self-feedback: compare (D), mRNA in the absence of Hb self-regulation (6B0H simulation of *hb* ^14F^, c.f. Figure 4), to (F), mRNA with self-regulation (WT 6B2H). Translation increases protein VMR about six-fold over mRNA, both without self-regulation (E) and with self-regulation (G). VMR is higher for both mRNA and protein with self-regulation, since the high protein VMR feeds back on mRNA transcription. Bcd noise has little effect on the *hb* mRNA or protein noise: the different VMRs point to different probability distributions (A vs. B and C), and there is negligible difference between simulations with Bcd noise (B, C) and without (F, G). I.e., the noise generated in transcription and translation dominates the noise transmitted from upstream regulator fluctuations.

Simulations of the Synthesis-Diffusion-Decay mechanism of Bcd patterning (Figure 2B) produce Poisson distributed noise, with a characteristic variance to mean ratio (VMR) equal to one: *n* = 6 stochastic simulations averaged VMR = 0.98 (averaged over all positions; Figure 5A shows a typical result; see also [25], [43]). (Distributions were generated from 1-second separated data points over 30 minutes of simulation during steady-state, *t* = 30–60 mins.) Though there are recent developments regarding the Bcd mechanism [44]–[46], these are not expected to strongly alter the Poissonian character of the noise. The Poisson distribution generally occurs for equilibrium fluctuations [47] and simple kinetic mechanisms [48], [49].

*hb* makes a nonlinear amplification of the Bcd signal which is no longer ‘simple’ kinetics: in the anterior region, *hb* mRNA shows a VMR 2–3 times that expected for a Poisson distribution (for *n* = 6 simulations, mean VMR for the 1–40%EL activated region was 2.4). Figure 5B shows typical *hb* mRNA VMR, for the same computation as Figure 5A. Translation produces further strong noise amplification for the protein, with mean VMR 16 times higher than Poisson (for 1–40%EL, *n* = 6). Figure 5C shows typical Hb protein VMR, for the same computation as Figures 5AB. (We model a typical [50], [36] translation rate of 35 proteins for each mRNA; higher or lower protein-to-mRNA ratio would give higher or lower protein VMR, respectively [see also 51 on this effect]. Non-Poisson noise amplification has previously been shown with translation in yeast [23]; our computations demonstrate the effect for spatially-distributed expression noise. The different probability distributions for Bcd, *hb* mRNA and Hb protein, evidenced by the increasing deviation from Poisson noise (e.g. Figure 5A to 5B to 5C), highlights the independent aspects of *hb* expression from the Bcd input signal.

*hb* self-regulation contributes to this non-Poisson noise. For *hb*-generated noise only (using a static Bcd gradient), simulations of *hb* ^14F^ (binding at 6 Bcd and 0 Hb sites, or ‘6B0H’) show a nearly Poisson VMR for the mRNA (mean VMR = 1.1, *n* = 6; Figure 5D is a typical result), less than half the VMR seen in WT (‘6B2H’) mRNA simulations (mean VMR = 2.3, *n* = 6; e.g. Figure 5F). In both 6B0H and 6B2H, translation increases protein VMR six-fold over mRNA (6B0H mean protein VMR = 6.2, *n* = 6, e.g. Figure 5E; 6B2H mean protein VMR = 14, *n* = 6, e.g. Figure 5G), but 6B2H starts from a higher mRNA VMR to produce a higher final protein VMR. Translation creates non-Poisson noise, so Hb protein is predicted to be non-Poisson with or without self-feedback. But WT protein is predicted to show a stronger deviation from Poisson than in *hb* ^14F^, since the self-feedback cycle creates non-Poisson noise at the transcriptional level for *hb* mRNA. (Other cases without self-feedback are expected to be like *hb* ^14F^: in simulations of the single Bcd site lacZ construct, mRNA VMR was also close to 1 with protein VMR close to 6.) It should be emphasized that while self-feedback increases the VMR, the overall noise is lower with self-feedback than without (Figure 3 vs. Figure 4; Table 1 vs. Table 2): self-feedback boosts production to higher mRNA and protein concentrations, which are overall less noisy.

For the concentration range measured for the Bcd-GFP gradient [4], Poisson fluctuations are very low, about 3–4% (relative standard deviation) at mid-embryo. At these levels there is very little difference between *hb* mRNA and protein noise levels in simulations with Bcd noise (e.g. Figures 5BC) or without Bcd noise (e.g. Figures 5FG). The following relations hold at 5% significance: *hb* mRNA and protein noise levels are correlated with each other, but neither are correlated with Bcd noise levels; mRNA noise levels show no difference in simulations with or without Bcd noise; protein noise levels appear slightly increased with Bcd noise; and the VMR trends discussed above show no difference with or without Bcd noise. While this does not rule out a minor effect from Bcd noise, the intrinsic noise arising from the kinetics of *hb* expression, especially translation and self-feedback, is expected to be a much greater factor than upstream Bcd fluctuations on overall *hb* noise. This is in contrast to the analysis in [4, eqn. 6] and [35, eqn. 15], in which nucleus-nucleus Hb noise was converted to Bcd input noise via assuming direct dependence of Hb output on Bcd input. By directly investigating the effect of the *hb* kinetics, our analysis indicates that this assumption is not likely to apply, especially at the low Bcd noise expected from its measured concentration [4]; rather, observed Hb noise is likely to be largely Bcd-independent. Experimental determination of probability distributions presents new technical and analytical challenges; the present simulations indicate the hallmarks of the non-Poisson distributions expected from transcription and translation, to guide such future work.

#### Bcd binding site number and binding strength affect expression noise

**Figure 6:**
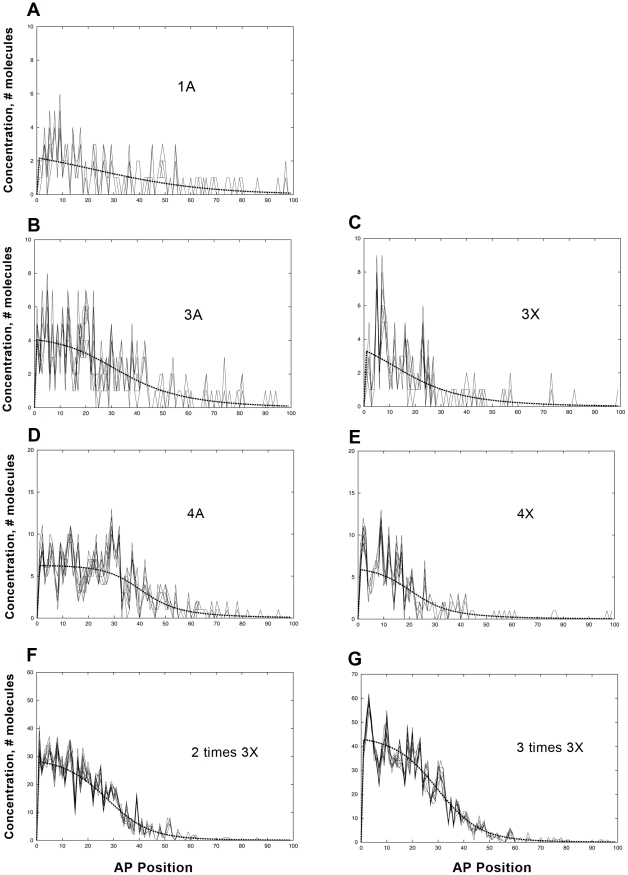
The effects of Bcd binding site number and strength on noise; simulations of lacZ constructs with Bcd binding sites only. (A) A single strong Bcd site (1A, pThb3 construct); very low and noisy expression, with much posterior activation (statistics for this Figure given in Table 3). (B) Three strong sites (3A, pThb10); expression is stronger and less noisy than (A). (C) Three weak Bcd sites (3X, pThb12); expression is noisier than (B). (D) Four strong sites (4A, pThb11); expression is stronger and less noisy than (B). (E) Four weak sites (4X, pThb13); expression is noisier than (D). (F) Doubling of the 3X promoter; noise is less than (C). (G) Tripling of the 3X promoter; lower noise again than (F). Noise decreases for increasing strength and increasing number of binding sites.

The subset of Driever lacZ constructs with only Bcd binding sites map out the degree to which transcription depends on Bcd binding (without Hb regulation). Model parameters were set (‘Experimental data determines model parameters’ section) to match expression levels and boundary positions in [34]; stochastic simulations predict the noise characteristics of the Bcd-dependent expression – these are summarized in Figure 6 and Table 3. Since these constructs are made in WT embryos, simulations include the full Hb model (Figure 2B), plus parallel reactions for production of lacZ and β-galactosidase from the binding sites appropriate to the construct. The 1A construct (pThb3, a single strong Bcd site) is at the limit of experimental detection [34]. The corresponding ‘on’ levels of mRNA in the model are on the order of 2 copies per nucleus (Figure 6A), producing very high noise (Table 3). This produces random activation along the length of the system and an indeterminate AP pattern. In constructs with 3 Bcd binding sites, anterior expression is observed to be more distinct. In addition to the increased expression with 3 sites, simulations predict that stronger binding, 3A (Figure 6B, pThb10) vs. 3X (Figure 6C, pThb12), decreases noise (Table 3). Adding a 4th Bcd site shows yet higher anterior expression, and the model again predicts lower noise for stronger binding, 4A (Figure 6D, pThb11) vs. 4X (Figure 6E, pThb13, Table 3). We predict that increasing number of sites also decreases noise. This is indicated by the decrease in noise from 1A to 3A to 4A (Figure 6 A to B to D), and also by simulations for constructs in which the 3X motif is multiplied: 2 times 3X (Figure 6F, pThb15) and 3 times 3X (Figure 6G, pThb16) show progressive reduction of noise from the single 3X (Figure 6C). Increasing binding sites or binding strength is associated with increased transcription, producing higher mRNA concentrations which are not as dominated by noise (binding site number and expression intensity are experimentally correlated, Table S1; for stochastic dynamics, stronger binding increases the bound, transcribing time; a recent study in yeast shows larger pulses of production with more binding sites [52]). The developmental effects of noise may also depend on pattern shape: as binding site number increases, the slope of the expression pattern sharpens, decreasing the positional effects of concentration fluctuations. In high noise situations, such as Figure 6A, there are two types of small number statistics: for DNA, the number of Bcd binding sites is small; and for mRNA, the numbers of copies produced per nucleus is also small. As the number of binding sites increases, the DNA source of the noise is diminished; as transcription is increased, the mRNA source of the noise is diminished. To summarize, the model predicts that noise decreases for stronger binding (4X to 4A; 3X to 3A) and for increased numbers of binding sites (1A to 3A to 4A; 3X to 2*x*3X to 3*x*3X).

**Table 3:** Noise levels, varying Bcd binding.

Model	1A	3A	3X	4A	4X	2*x*(3X)	3*x*(3X)
	(Fig. 6A)	(Fig. 6B)	(Fig. 6C)	(Fig. 6D)	(Fig. 6E)	(Fig. 6F)	(Fig. 6G)
	82%	64%	82%	47%	102%	48%	30%

For the simulations shown in Figure 6.

#### The effect of Hb binding sites on expression noise

**Figure 7 pcbi-1001069-g007:**
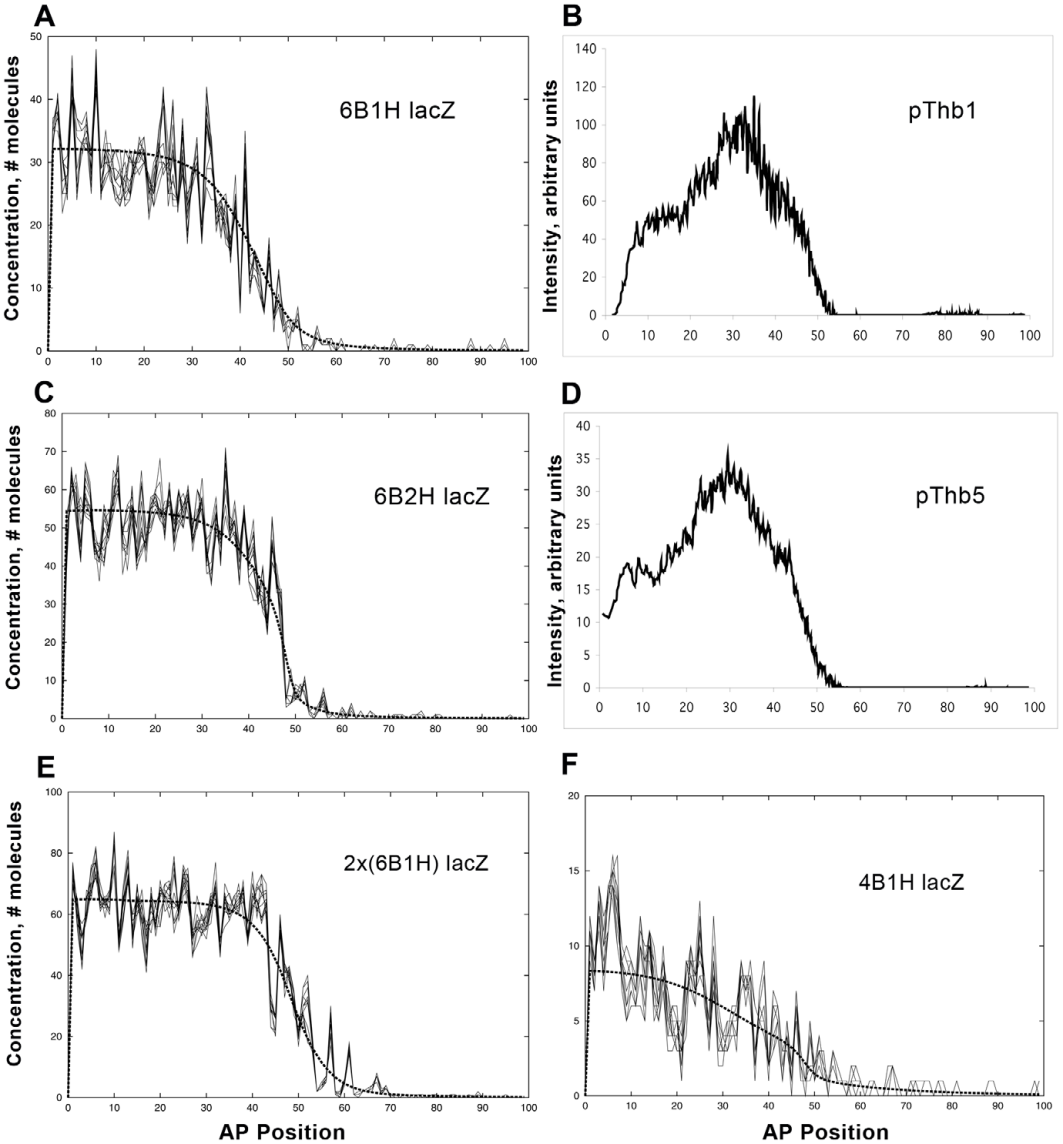
The effects of Hb binding site number and strength on noise. (A) Simulation of lacZ expression from 6 Bcd sites (3A3X) and one Hb site (in a WT background), compare to (B) lacZ expression in an embryo with the pThb1 promoter construct. (C) Simulation with 6 Bcd sites and 2 Hb sites, compare to (D) lacZ in a pThb5 embryo (promoter indicated by green arrows in Figure 2A). Comparison of (C, D) and (A, B) indicates that the 2^nd^ Hb increases expression and slope and may slightly decrease noise. Noise statistics for this Figure are in Table 4. (E) Simulation of the pThb8 construct, a doubled 6B1H sequence. (F) Simulation of the pThb2 construct, which has a shorter fragment of the *hb* promoter, with 1 Hb site and 4 Bcd sites (2A2X1H); the model predicts increased noise for this truncated promoter.

In addition to the WT-*hb* ^14F^ comparison above, lacZ constructs with Hb binding sites shed some light on the role of Hb in noise control. pThb1 has 6 Bcd sites (3A3X), but only one Hb site (6B1H). A simulation is shown in Figure 7A; lacZ for a pThb1 embryo is shown in Figure 7B. The pThb5 construct is driven by the 3A3X Bcd sites and 2 Hb sites, the core of the WT proximal promoter (green lines, Figure 2A): Figure 7C shows a simulation; Figure 7D shows lacZ for a pThb5 embryo. The simulations suggest a slight drop in noise with addition of the 2^nd^ Hb (statistics in Table 4), and the experimental data support this. The first Hb binding has a minor effect (reflected in its minor posterior shift compared to *hb* ^14F^). Binding of the 2^nd^ Hb in the model increases expression and creates a sharper boundary. In the pThb8 construct the 6B1H promoter (Figures 7A, B) is doubled; simulating this (Figure 7E) doubles production and decreases noise (to similar levels to pThb5; but the boundary is not sharp in the absence of the 2^nd^ Hb site). Finally, for the pThb2 construct, with 4 Bcd sites (2A2X) and 1 Hb site, we predict (simulation, Figure 7F) a loss of determinacy and an anterior shift compared to 6B1H (Figure 7A). The expression and noise (Table 4) are comparable to the 4A construct (Figure 6D, Table 3), suggesting the 1 Hb site may compensate for the two weak X sites in pThb2.

**Table 4:** Noise levels, varying Hb binding.

Model	3A3X1H	3A3X2H	2*x*(3A3X1H)	2A2X1H
	(Figure 7A)	(Figure 7C)	(Figure 7E)	(Figure 7F)
	24%	16%	16%	47%

Experiment	pThb1^a^	pThb5^b^
	(Figure 7B)	(Figure 7D)
	8.0 (0.75) %	6.3 (2.5) %

For the simulations and embryos shown in Figure 7.

a Average (std. dev. in brackets), *n* = 2.

b Average (std. dev. in brackets), *n* = 3.

#### Evolution

In *Drosophila*, highly-conserved domains exist within the *hb* promoter across at least 7 species (found with the EDGI server [53]). The *hb* promoter is also a well conserved motif for studying the evolution of early AP patterning across flies [54],[[55]; Wunderlich et al., 50^th^ *Drosophila* Conference Proceedings, p. 74]. It has been shown that Bcd strong (A) and weak (X) sites are found across several species of flies, but that the number of sites varies (Table 5). These varied promoters all create long-germ band type Hb patterns, dividing the embryo roughly into anterior and posterior halves. Therefore, a model of Hb patterning should be robust to forming WT pattern over the natural variation seen across these species, from 4 Bcd sites in *Drosophila virilis* to 10 Bcd sites in *Musca domestica*. In our model, we have altered the Bcd binding as described in Table 5, adding or removing Bcd sites from the WT *D. melanogaster* model (Figure 2). We have modelled other species as having 2 Hb binding sites and early Hb pattern as in *D. melanogaster*. The model is robust to this degree of cross-species variability. We predict slight posterior shifts for species with more binding sites than *D. melanogaster*. With 4 binding sites the model predicts some anterior shifting of the mid-embryo border and loss of expression in the anterior-most regions; this latter depends on the relative contributions of Bcd and early Hb. Noise levels are predicted to be somewhat higher for *D. virilis*, with 4 Bcd sites, but the other species' promoters should produce similar noise levels to *D. melanogaster* (Table 5).

**Table 5:** Simulation results, other flies.

Bcd sites^a^	3A,1X	3A,4X	5A,4X	6A,4X
***Species***	***Drosophila virilis***	***Lucilia***	***Calliphora***	***Musca***
**Position of boundary** (%EL)	43	48	49	49
**Sharpness** (degrees)	85	83	83	83
**Bcd binding**	WT *melanogaster* 3A, plus 4^th^ Bcd binds as X_4_ (see Table S2).	WT 3A3X, plus 7^th^ Bcd binds as X_6_.	As *Lucilia*, plus 8^th^ & 9^th^ Bcds as A_3_.	As *Calliphora*, plus 10^th^ Bcd as A_3_.
**Noise level**: protein	18%	4.9%	5.2%	4.4%
mRNA	24%	13%	11%	13%

a Numbers of strong (A) and weak (X) Bcd binding sites from [54], [55]. All simulations run with *D. melanogaster* WT 2Hb sites and early Hb.

#### Variability between DNA copies

**Figure 8:**
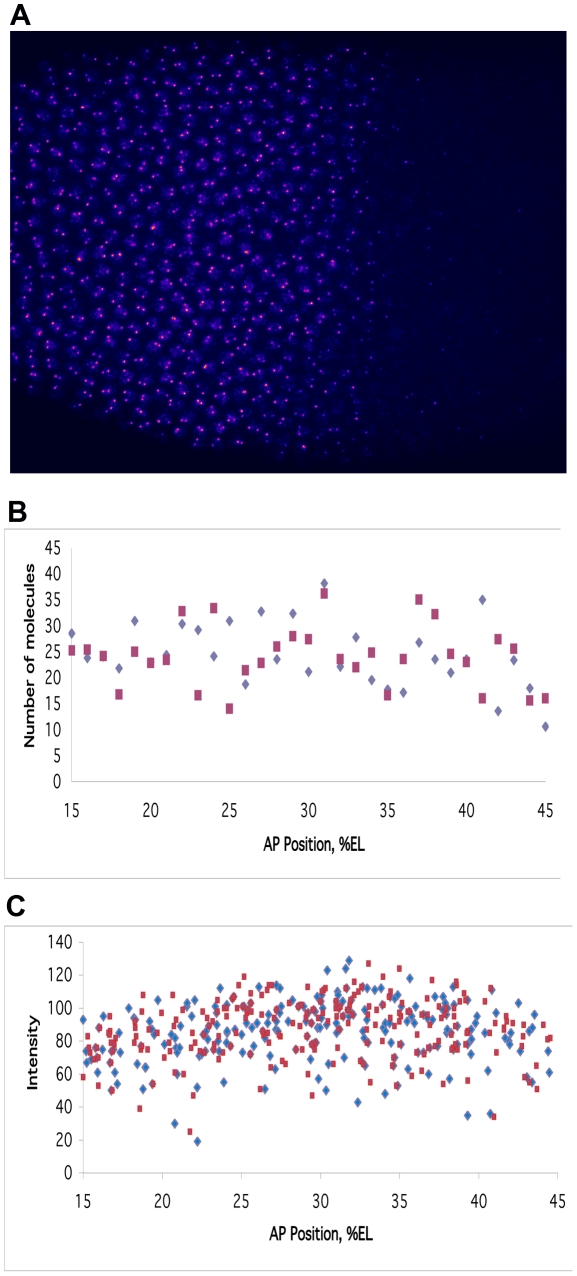
Variation within nuclei, transcription from two copies of the promoter. (A) lacZ mRNA labelling (pink) at nuclear dot resolution in a pThb5 embryo (nuclei in blue), 30–36 minutes into cleavage cycle 14 (c.f. Figure 7D). (B) Computed lacZ mRNA levels, for two equal and independent promoters, A (blue) and B (red), at 30 minutes; the simulation shown has a relative noise between A and B of 31%, an average value (see Table 6 for statistics). (C) Comparable plot of intensity against AP position for the embryo in (A), A–B dot pairs are coloured as in (B). This data has A–B relative noise of 17%.

Experimental resolution is reaching the level to visualize transcripts coming off the different copies of a gene within each nucleus (nuclear dots [56]; Figure 8A). Such data holds the best promise for measuring transcriptional/translational noise, distinct from noise generated by other sources (e.g. transport). We simulated transcription occurring at two independent promoters (A and B) per nucleus, with the resulting mRNA being translated into a pooled protein. This was done for WT (6B2H), the *hb* ^14F^ (6B0H) mutant, and pThb5 lacZ expression (shown in Figure 8B). Figure 8C shows mRNA intensity per nuclear dot for the pThb5 embryo in Figure 8A, for comparison. The model predicts that *hb* ^14F^ and WT mRNA should have higher and lower variability than pThb5 lacZ, respectively: variability decreases from 6B0H to pThb5 to 6B2H, with no overlap between sets of simulations (Table 6; predictions, as above, are for relative trends, not absolute experimental values). For 6B0H simulations (*hb* ^14F^), the (pooled) Hb protein does not feed back on transcription to synchronize the A and B promoters; any relation between A and B is due to the shared Bcd input signal. For 6B2H simulations (WT), feedback of the pooled Hb protein onto the promoters provides an averaged signal which decreases the variability between A and B transcription. The intermediate variability of pThb5 reflects that the pooled Hb binds the lacZ-expressing promoters, but there is no self-feedback of the lacZ protein (β-galactosidase) on these promoters. The influence of self-feedback on reducing noise by boosting concentration was discussed above; here we predict that self-feedback also reduces variability from independent transcription at multiple promoter copies.

**Table 6:** Noise for simulations with two independent promoters per nucleus.

6B0H (*hb* ^14F^)	lacZ (pThb5)	6B2H (WT)
46–60%, avg. = 49%	24–35%, avg. = 30%	16–20%, avg. = 18%

Noise is calculated by 
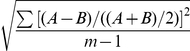
, where A, B are the mRNA at each transcription site, for 15–45%EL. *n* = 6 for each group of simulations.

## Discussion

The Bcd-Hb system has received a great deal of study over several decades, making it one of the best characterized systems for understanding the mechanistic details of positional specification by gradient reading. We have used binding site information for the *hb* promoter in conjunction with quantitative imaging to develop and test a stochastic model of expression dynamics. This has allowed us to characterize the noise inherent in gene expression due to the low copy number of DNA (numbers of promoters and binding sites) and mRNA. Identifying how noise is controlled in spatial gene expression patterns is a fundamental problem; the dynamics at the *hb* promoter provide a model for how this might occur in many patterning events. Modelling the variability in the data, in addition to modelling average features, provides an extra experimental dimension for refining and validating models of gene regulation.

Prior work has focused on the effects of Bcd noise on Hb [35]. However, we experimentally observe statistically significant noise reduction in the process of translation and due to Hb self-feedback, which validate model predictions: our study points to the critical aspects of intrinsic noise arising in Hb production, independent of external fluctuations. For the noise levels associated with Bcd's measured concentration [4], we see little, if any, effect of Bcd concentration fluctuations on *hb* production. External noise is overshadowed by intrinsic noise arising in binding site occupancy and modulated through transcription and translation. Work in [36] indicated that promoter occupancy noise could be reduced by fast Hb diffusion. However, the steep angle of the Hb boundary suggests a slow diffusivity not optimized for noise reduction. Our work suggests several mechanisms in the *hb* dynamics which reduce noise.

In the absence of Hb self-feedback, as in the *hb* ^14F^ mutant and the lacZ reporter constructs, output is noisier - less shielded from the noise of binding site occupancy. Fitting the binding strengths and production rates associated with 6 individual Bcd sites and 2 Hb sites to the lacZ data has enabled us to predict the degree to which increasing number and strength of binding sites (Bcd and Hb) can buffer promoter occupancy noise. Such basic noise reduction may have evolved fairly independently of other mechanisms; our computations suggest *hb* noise is similarly controlled in flies with between 4 and 10 Bcd sites.

Self-feedback is a major component of WT Hb expression [1], responsible for large changes in protein production and boundary sharpness from *hb^14F^* to WT [30]. We observe a significant effect of self-feedback in reducing protein noise levels (Figures 3C vs. 4A; Tables 1 and 2). Our calculations indicate self-feedback is responsible for a change in anterior mRNA levels from roughly 30 (*hb* ^14F^, Figure 4C) to roughly 200 copies per nucleus (WT, Figure 3B), from a noise-dominated to a more deterministic regime [29], which in turn reduces translational variability in WT. In addition, we predict that self-feedback reduces variability arising from transcription at multiple promoters within nuclei (Table 6).

We predict that the noise reduction seen with translation (Figure 3C vs. 3D, Table 1) is similarly due to a concentration difference, since one copy of mRNA makes multiple copies of protein (Figure 3A vs. 3B; Figure 4B vs. 4C; Tables 1 and 2). Translation is expected to produce non-Poisson *hb* probability distributions, distinct from the Poisson noise expected for Bcd. Such non-Poisson ‘bursting’ noise has been characterized in yeast [23], in which protein variance scales with mean concentration but with a VMR far greater than for a Poisson process, due to amplification of noisy low mRNA copy number. For *hb*, we expect deviations from Poisson to be stronger in protein than mRNA, and strongest with self-feedback than without. The observation of lower relative noise in WT protein than either WT mRNA or *hb* ^14F^ protein suggests that its higher concentration overcomes the non-Poisson bursting effects. (A purely Poisson-distributed WT Hb would be expected to have even lower noise and show larger differences with both WT mRNA and *hb* ^14F^ protein.)

Published data [4], [30], [32], [34], [38], [41], [57] places distinct constraints on any model of the *hb* promoter, for rates of regulator binding and production. Conclusions can be made from these regarding the relative values of these constants (e.g. relative WT and *hb* ^14F^ Hb levels indicate that WT production rates are 7 times higher; early vs. mid-cycle 14 data indicates Hb production needs to increase concentration by about 50% in 30 minutes), and these conclusions can be followed through for their implications for relative noise differences. If absolute concentrations were higher or lower than estimated, absolute noise levels would be lower or higher, respectively; but the relative results would hold. We have made noise predictions at energid (nucleus plus associated cytoplasm) resolution for mRNA and protein output for: WT; the *hb^14F^* mutant; 11 lacZ reporting constructs with various combinations of binding sites; and 4 other species of flies. Enhancing our confidence in the model, we have corroborated model predictions at this resolution for: higher mRNA than protein noise; higher *hb^14F^* than WT noise; and the indication of noise decrease due to the 2^nd^ Hb site, comparing the pThb1 and pThb5 constructs. In addition to these, the model illustrates the reduction in noise available from increasing binding site number and strength; the role of moderate cooperativity and slow protein timescales for limiting noise; and the degree to which within-embryo noise can generate between-embryo variability. These results indicate the degree to which *hb* noise amplitudes are determined by expression dynamics, and how these dynamics produce *hb* probability distributions distinct from Bcd's.

We have also taken data at intra-nuclear resolution, imaging transcript production from different copies of the promoter within each nucleus. This is at current technical limits of spatial resolution (though recent very high resolution studies with GFP [58], [59] are promising for *bcd*). Data analysis and modelling at this level shows promise for separating transcriptional noise from other types, such as from inter-nuclear transport. (See [60] for a recent study showing the effect of promoter state on pattern synchrony at this degree of resolution.)

Only Bcd and Hb regulation, at the specified binding sites, are considered in the model. The dynamics of this core promoter region reproduce many of the deterministic and stochastic features of Hb activation in the anterior region. Additional Hb sites, such as those in the distal P1 promoter, are known to affect later, posterior expression – any effect of these sites in early expression would be incorporated into the 2 Hb sites of the current model. Additional Bcd sites would not be expected to greatly influence expression, based on *Musca* and the simulations with up to 10 Bcd sites. The positioning of final expression patterns, especially later in cycle 14, do depend on other factors, such as inhibition by other gap genes (e.g. [61]). (Head gap genes are likely involved in the lower Hb expression at 0–15%EL, e.g. Figure 3C [15].) The region of 40–50%EL, where the Hb boundary interprets a marginal decrease in the Bcd gradient, is promising for quantifying these factors. In preliminary computations, reduced or missing initial Hb gave reduced expression in this parasegment 4 region. Similar phenotypes are observed with some gap mutations [62]. Incorporation of other regulators in the model would permit exploration of their relative contributions to Hb noise in this region.

Hb itself forms a morphogenetic gradient, controlling the expression of a number of other gap genes in early segmentation, potentially at higher precision than Bcd [63]. The mechanism of secondary (Hb) gradient formation is in contrast to those for the primary (maternal) gradients, which do not form by spatially-distributed gene expression. The present work, therefore, has focused on how transcription and translation kinetics can be controlled to provide a determinate and precise secondary gradient for specification of the segmentation patterns. While our study has shown the importance of the details of promoter structure and expression dynamics (such as self-feedback) on *hb*'s expression noise, many of the noise motifs found here will be applicable to other genes (for instance, dependence on binding site number, non-Poisson amplification in transcription and translation). In this way, characterizing *hb* noise serves as a model for how zygotic gene expression gives rise to the determinate and reliable expression patterns underlying development.

## Methods

### Simulations

The model in Figure 2B was computed with the MesoRD software ([27]; http://mesord.sourceforge.net). This package allows deterministic (used for parameter fitting) or stochastic (used for noise prediction) solution of mechanisms involving reaction and diffusion. A kinetic scheme is entered as elementary reactions (Figure 2B), and rate constants (values in Tables S1, S2, S3) and diffusivities (for Hb and Bcd proteins, see Tables S2, S3 for values) are specified for the model species. Geometry was specified as a one-dimensional series of 100 subvolumes (each a 5µm cube), corresponding to the energids (nucleus plus cytoplasmic neighbourhood) along the AP axis. Computations solve for model species densities in each subvolume, according to the specified reactions and between-subvolume diffusion. In deterministic simulations (for parameter searches), we used a 4^th^ order Runge-Kutta solution method. For matching to data, boundary position and sharpness were determined at half-maximal concentration. For stochastic solutions, MesoRD solves the reaction-diffusion master equation, in which each reaction and diffusion event has a probability (set by the macroscopic rate constants) of occurring in a unit of time. The software implements the next subvolume queuing method [26], [27], [64] to significantly improve memory and processing requirements, making computation possible for the number of species and subvolumes in the *hb* model. All computations are run in real units (µm, s, etc.).

### Parameter determination

Model parameters were determined by fitting macroscopic features of published data: boundary position and angle, expression levels, and timescales. As described in the Results, building up from the lacZ data to *hb* ^14F^ and WT constrains the values of the binding rates and diffusivities in the model. Further details are given in Text S1.

### Experimental data

Fly stocks, staining and imaging were as in [30]. Whole mount embryos were imaged by laser confocal scanning microscopy, from WT Oregon-R, *hb^14F^*, and the lacZ construct (pThb1,5; [34]) lines. All embryos were heat fixed and immunostained for Hb protein. Fluorescent in situ hybridization (FISH) was used for mRNA determination, for *hb* and for lacZ, following the method of [65]. Images were collected using an HC PL APO 206 objective and variable digital zoom (1.2–1.56). Fluorophores were excited by laser at different wavelengths (488, 555, and 647 nm), and detected via a filterless spectral separation system. Channels were scanned sequentially. The microscope was set so that maximum expression was 255 on an 8-bit scale. To reduce photomultiplier noise, each image was scanned sequentially 16 times and the results averaged.

### Image processing

Raw images from the confocal microscope, 1024×1024 pixels, were cropped and rotated for standardization. Each energid (each nucleus plus its cytoplasmic neighbourhood) was identified by Voronoi tessellation [66]. See Text S2 for details (Figures S1, S2). Averaged pixel intensities within each energid (Figure S3) were used for comparison to simulation output. Data was used from a 10% DV (dorsoventral) strip, centred on the AP midline, in order to minimize geometric distortion from the embryo periphery. Background fluorescence for these lateral images follows a half ellipsoid (c.f. [67]). We found the parameters of the ellipsoid for each image by a Genetic Algorithms technique (c.f. [68]). An initial visual inspection of the data for each embryo was needed to estimate the approximate height of the background. The quantitative measure of fitting quality for a given set of parameters was based on minimizing the distance between the data points and the ellipsoid surface. Previous approaches used preliminary statistical analysis of the dynamics and positioning of areas of zero specific signal (i.e. areas where all fluorescence was background), requiring large datasets [67]. Our supervised evolutionary search gives a much more efficient method for directly analyzing each image.

### Statistics

Singular Spectrum Analysis (SSA) [69], a non-parametric technique with an adaptive filter, has been recently used for separating confocal intensity data into components [70], [71]. Its extension, 2D-SSA [72], was applied to the 2D (AP and DV) intensity surface; the leading components of the decomposition give the pattern's trend. Noise was then quantified from the difference of each energid's intensity to the trend value at each position (i.e. local residuals). See Text S3 (Figures S4, S5, S6, S7, S8, S9, S10, S11, and S12) for more detail on the SSA data analysis. Noise measures were calculated from the anterior expressing regions (15–45% EL) as 
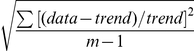
, where *data* is the average pixel intensity for an energid and *trend* is the SSA-extracted trend at that position (i.e. the noise measure is a standard deviation for the relative residuals). Noise was calculated similarly for simulation output, from the difference of the stochastic output and the deterministic solution at each position (see Table 1 footnote).

## Supporting Information

Figure S1 Nuclear identification of a single WT embryo probed for the Hb protein (same embryo and data as Figure 3CD in the main text). Image processing routines were developed in order to identify the nuclei of the embryos. Protein data are used at this stage, due to the clear visualization of the nuclei, with the drawback that regions where the protein is not expressed cannot be detected (such as in the posterior of this image).
(0.06 MB PDF)

Figure S2 Energid identification. Application of the generalized Voronoi diagram to the image in Figure S1 (WT embryo, Hb protein). Blue mesh shows the energid boundaries identified by the Voronoi diagram, overlying the original Hb protein image.
(0.09 MB PDF)

Figure S3 Visualization of the quantified protein and mRNA patterns. Dots (centred on the energids) are colourmapped by the average pixel intensity of each energid. (A) WT embryo, Hb protein (same data as Figure 3C in main text). (B) WT embryo, hb mRNA (same data as Figure 3D in main text). (C) Embryo with the pThb5 construct, lacZ mRNA expression (same data as Figure 7D in main text).
(0.12 MB PDF)

Figure S4 SSA fitting - nuclear centres and cropping rectangle.
(0.04 MB PDF)

Figure S5 SSA fitting - nuclear centres and regular interpolation grid.
(0.03 MB PDF)

Figure S6 SSA fitting - regularized data.
(0.08 MB PDF)

Figure S7 SSA fitting - trend on the regular grid.
(0.03 MB PDF)

Figure S8 SSA fitting - W-correlations for window 33×33 (black - 1.0, white - 0.0).
(0.02 MB PDF)

Figure S9 SSA fitting - effect of window size. AP data (blue) and trend (black). Trend is along the AP axis, and expression is from a 15% DV wide strip around this.
(0.03 MB PDF)

Figure S10 SSA fitting - effect of number of components. Trend and data along the AP axis. Trend is given by 2 components (c.f. 3 components in Figure S9).
(0.03 MB PDF)

Figure S11 SSA fitting - residual plots, for different numbers of components, in 15% wide strip around the AP axis.
(0.03 MB PDF)

Figure S12 SSA fitting - noise vs. trend, with moving statistics (left - absolute, right - relative), showing multiplicative noise.
(0.03 MB PDF)

Table S1 Relative intensities in different experiments set production rates.
(0.03 MB PDF)

Table S2 Bcd binding strengths.
(0.04 MB PDF)

Table S3 Hb binding strengths.
(0.04 MB PDF)

Text S1 Deterministic modelling and experimental constraints set parameters.
(0.05 MB PDF)

Text S2 Image processing and analysis.
(0.02 MB PDF)

Text S3 Trend extraction with 2D-SSA.
(0.03 MB PDF)
