# Supplementary figures and images for: Gene Expression Noise in Spatial Patterning: hunchback Promoter Structure Affects Noise Amplitude and Distribution in Drosophila Segmentation
Source: PLoS Comput Biol. 2011 Feb 3;7(2):e1001069. doi: 10.1371/journal.pcbi.1001069 (PMC3033364; doi:10.1371/journal.pcbi.1001069)

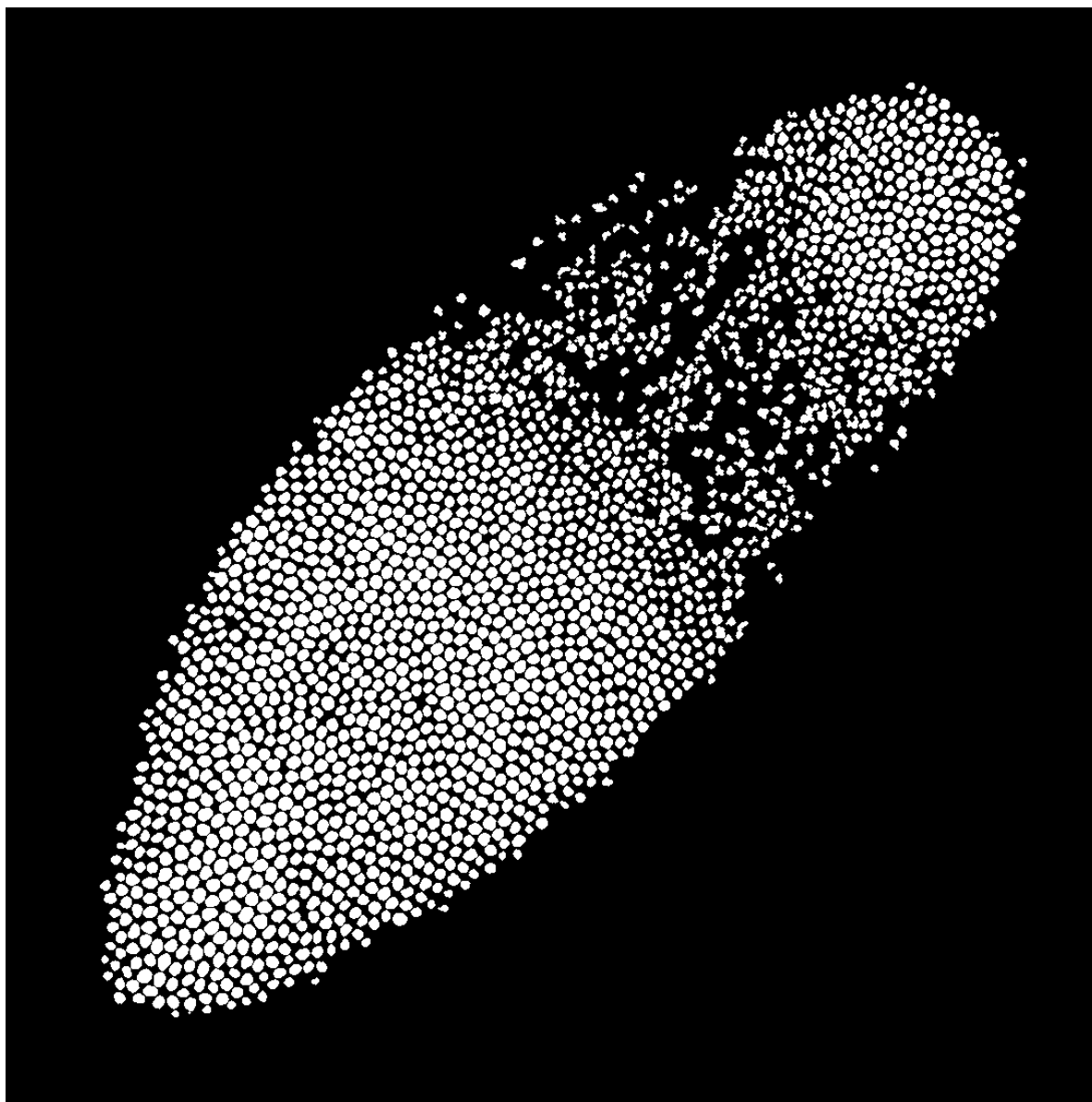

Supplement: Figure S1 — Nuclear identification of a single WT embryo probed for the Hb protein (same embryo and data as Figure 3CD in the main text). Image processing routines were developed in order to identify the nuclei of the embryos. Protein data are used at this stage, due to the clear visualization of the nuclei, with the drawback that regions where the protein is not expressed cannot be detected (such as in the posterior of this image). (0.06 MB PDF) [file pcbi.1001069.s001.pdf]

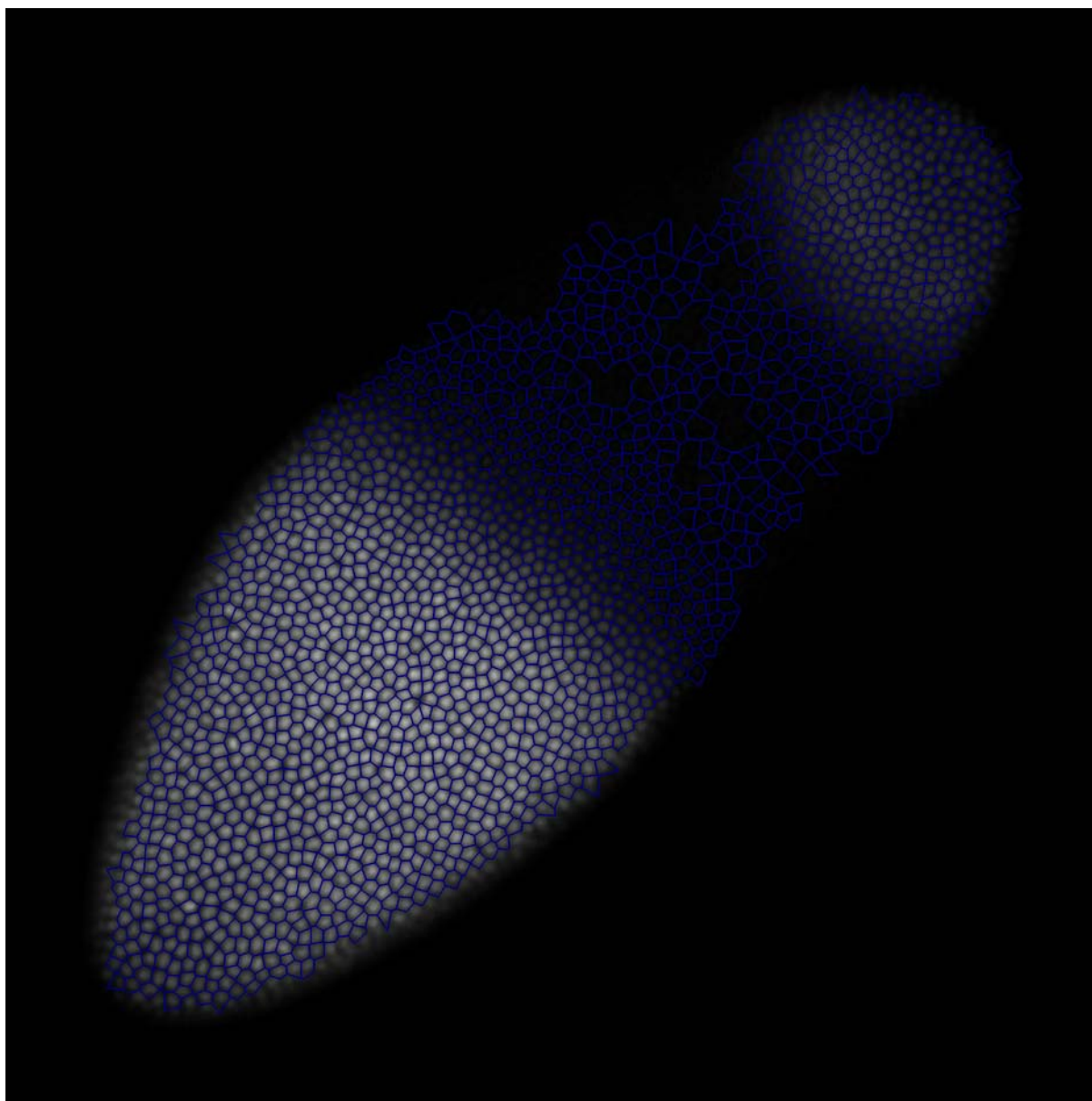

Supplement: Figure S2 — Energid identification. Application of the generalized Voronoi diagram to the image in Figure S1 (WT embryo, Hb protein). Blue mesh shows the energid boundaries identified by the Voronoi diagram, overlying the original Hb protein image. (0.09 MB PDF) [file pcbi.1001069.s002.pdf]

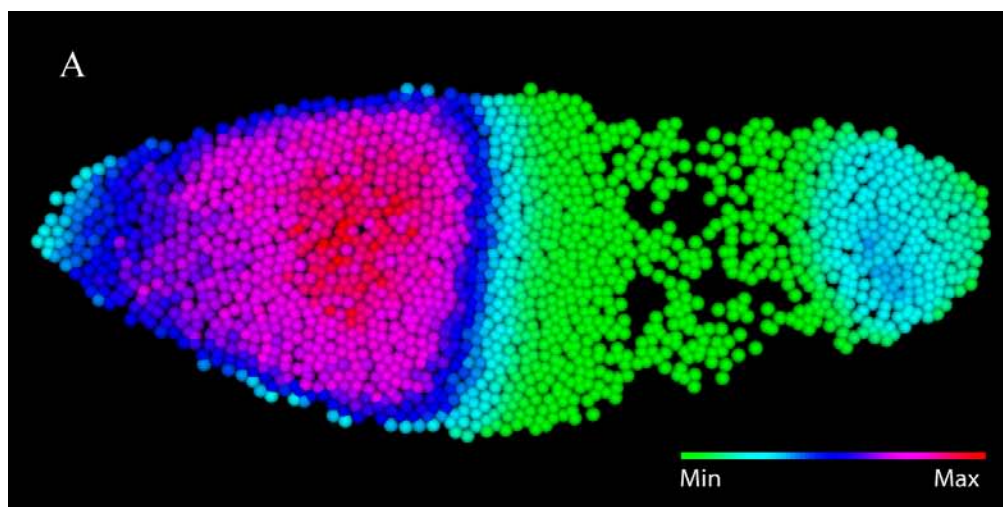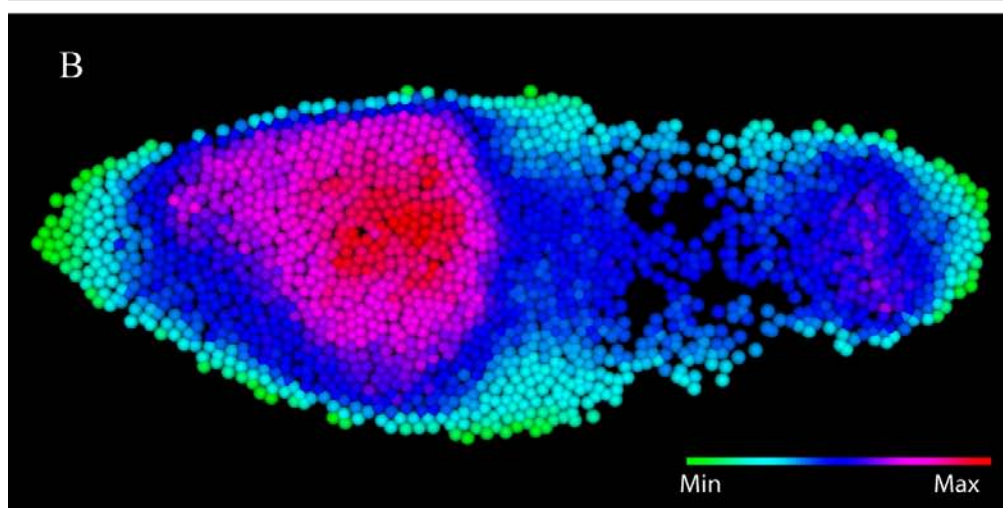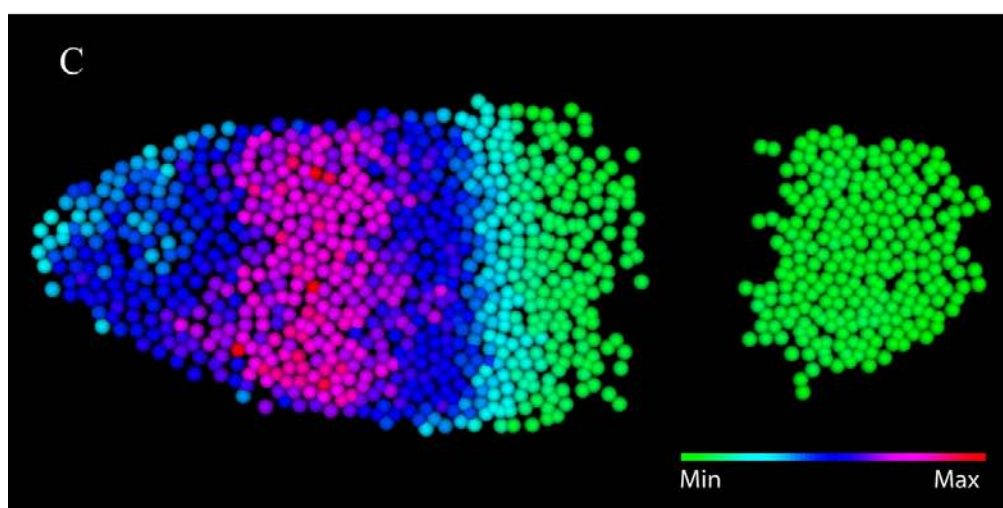

Supplement: Figure S3 — Visualization of the quantified protein and mRNA patterns. Dots (centred on the energids) are colourmapped by the average pixel intensity of each energid. (A) WT embryo, Hb protein (same data as Figure 3C in main text). (B) WT embryo, hb mRNA (same data as Figure 3D in main text). (C) Embryo with the pThb5 construct, lacZ mRNA expression (same data as Figure 7D in main text). (0.12 MB PDF) [file pcbi.1001069.s003.pdf]

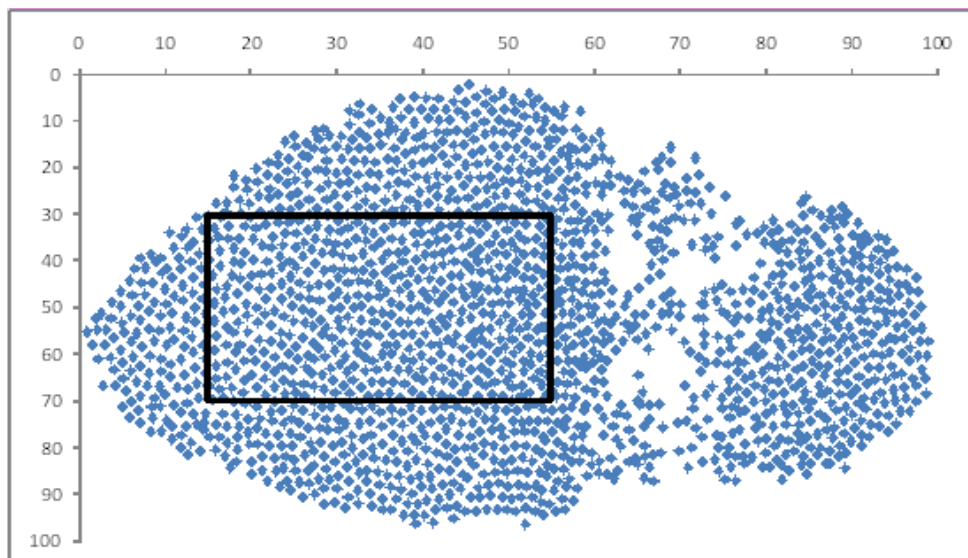

Supplement: Figure S4 — SSA fitting - nuclear centres and cropping rectangle. (0.04 MB PDF) [file pcbi.1001069.s004.pdf]

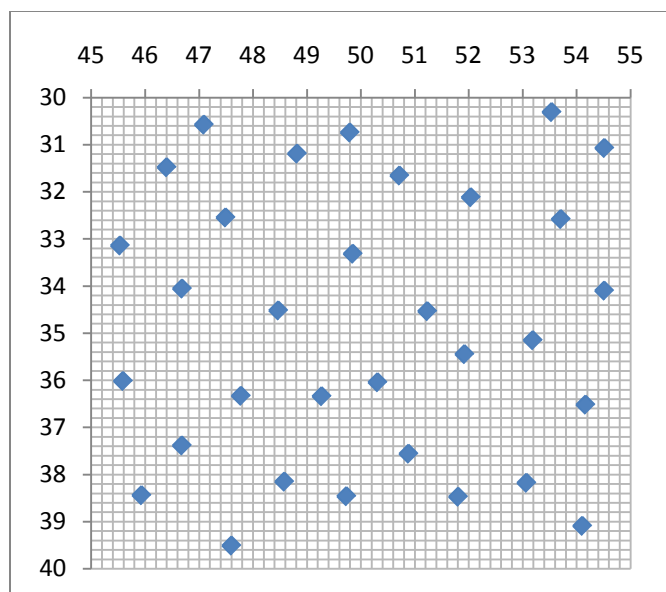

Supplement: Figure S5 — SSA fitting - nuclear centres and regular interpolation grid. (0.03 MB PDF) [file pcbi.1001069.s005.pdf]

Emb\_12\_Image010\_Protein\_Emb\_12\_Image010\_Protein.lacZ.bgr\_reg.dat

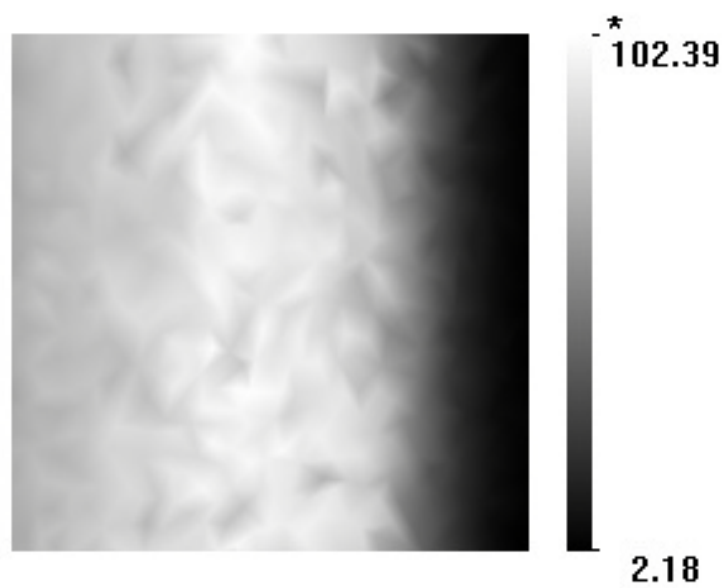

Supplement: Figure S6 — SSA fitting - regularized data. (0.08 MB PDF) [file pcbi.1001069.s006.pdf]

Emb\_12\_Image010\_Protein\_Emb\_12\_Image010\_Protein.lacZ.bgr\_reg.dat,

**#1, Components (1, 2, 3)**

(Lx, Ly) = (33, 33)

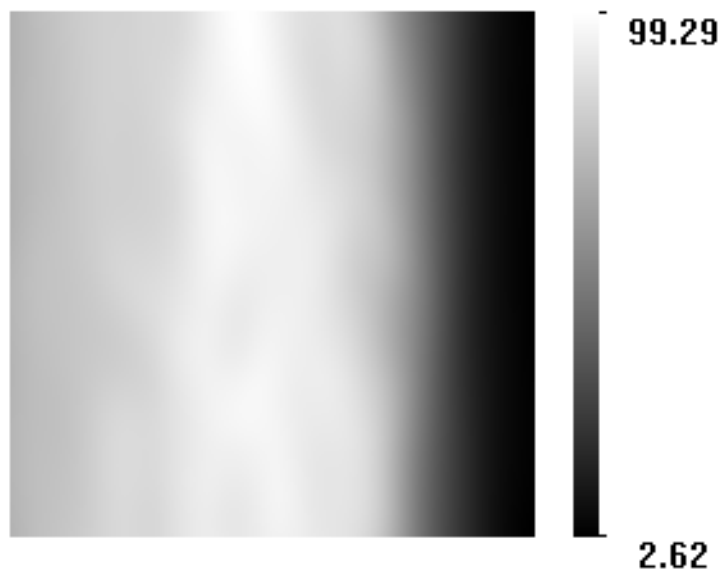

Supplement: Figure S7 — SSA fitting - trend on the regular grid. (0.03 MB PDF) [file pcbi.1001069.s007.pdf]

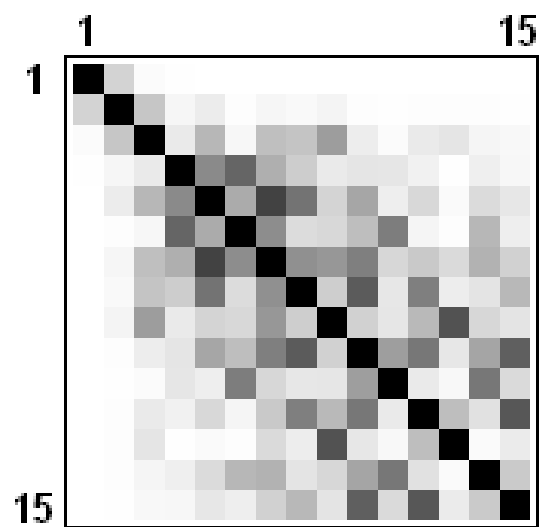

Supplement: Figure S8 — SSA fitting - W-correlations for window 33×33 (black - 1.0, white - 0.0). (0.02 MB PDF) [file pcbi.1001069.s008.pdf]

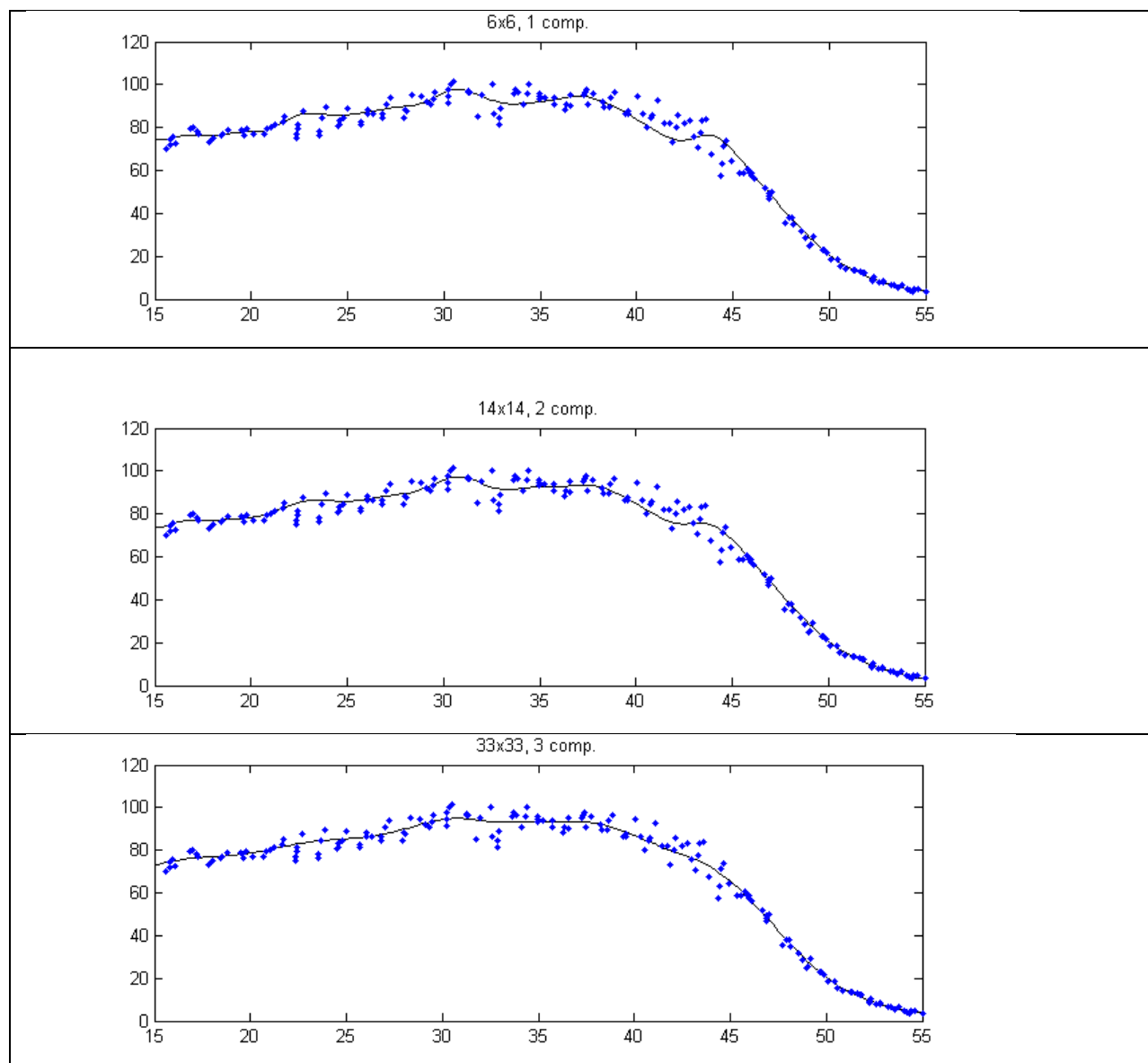

Supplement: Figure S9 — SSA fitting - effect of window size. AP data (blue) and trend (black). Trend is along the AP axis, and expression is from a 15% DV wide strip around this. (0.03 MB PDF) [file pcbi.1001069.s009.pdf]

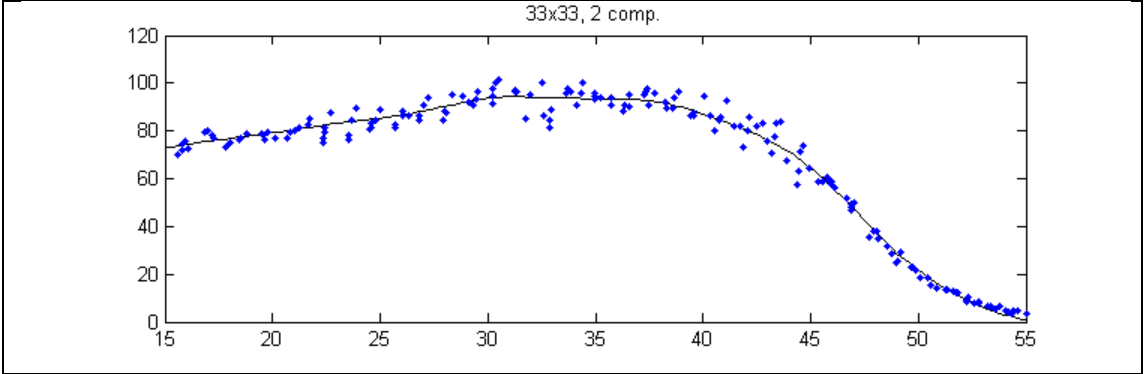

Supplement: Figure S10 — SSA fitting - effect of number of components. Trend and data along the AP axis. Trend is given by 2 components (c.f. 3 components in Figure S9). (0.03 MB PDF) [file pcbi.1001069.s010.pdf]

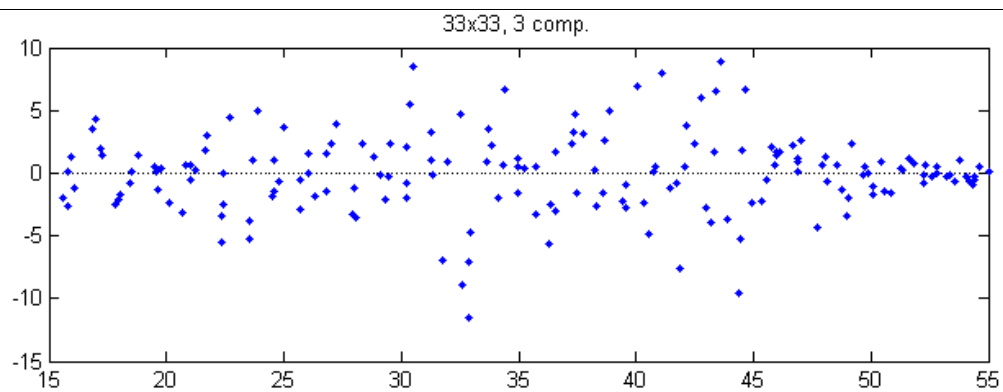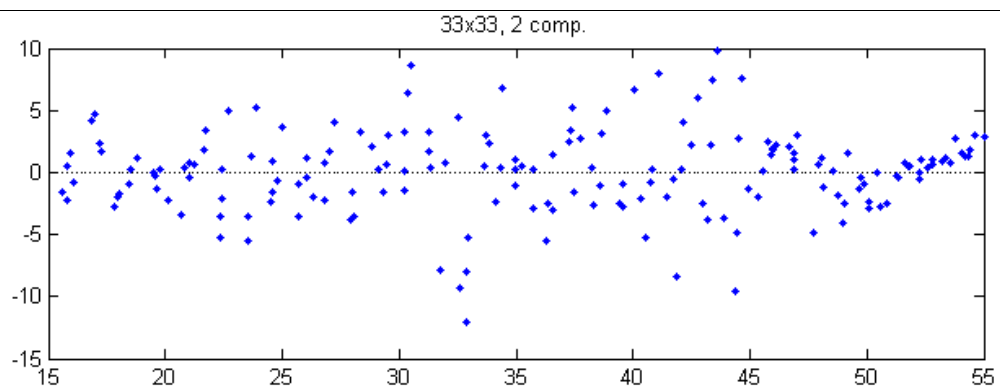

Supplement: Figure S11 — SSA fitting - residual plots, for different numbers of components, in 15% wide strip around the AP axis. (0.03 MB PDF) [file pcbi.1001069.s011.pdf]

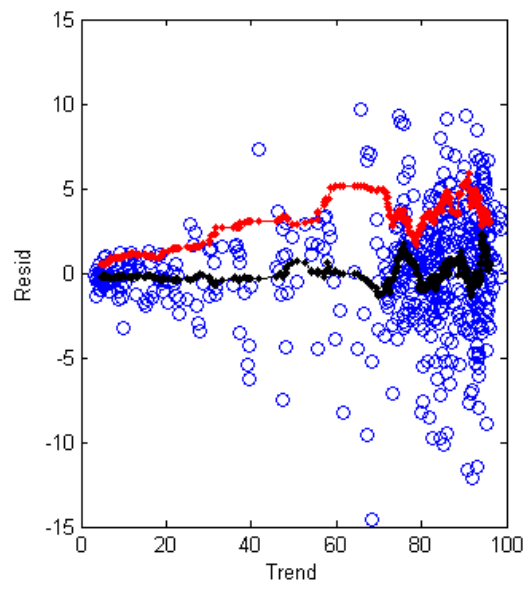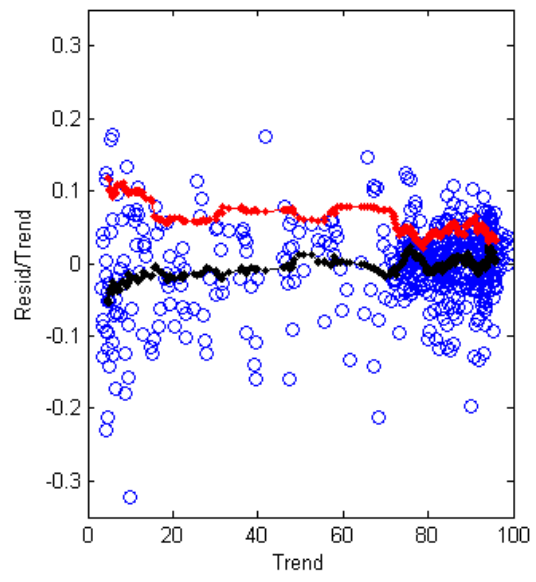

Supplement: Figure S12 — SSA fitting - noise vs. trend, with moving statistics (left - absolute, right - relative), showing multiplicative noise. (0.03 MB PDF) [file pcbi.1001069.s012.pdf]
