# Supplementary material for: Gene Expression Noise in Spatial Patterning: hunchback Promoter Structure Affects Noise Amplitude and Distribution in Drosophila Segmentation
Source: PLoS Comput Biol. 2011 Feb 3;7(2):e1001069. doi: 10.1371/journal.pcbi.1001069 (PMC3033364; doi:10.1371/journal.pcbi.1001069)
Supplement: Table S1 — Relative intensities in different experiments set production rates. (0.03 MB PDF) [file pcbi.1001069.s013.pdf]

Table S1: **Relative intensities in different cases set production rates.**

**Number of Sites**<br>(XBcd,YHb)	**Protein Concentration**<br>(max, in molecules/nucleus)[a]	**Transcription rates**<br>(s^{-1})
**6B,2H**<br>*WT promoter*	**7000**	1st Hb: k_2=1e-1<br>2nd Hb: k_5=3.1e0
**6B,0H**<br>hb^{14F}	**1050**<br>(hb^{14F} is 15% of WT expression [30]; “Strong” from [34])	k_{22}, k_{25}=3.6e-1[b]
**4B,0H**<br>*pThb11,13* [c]	**210**<br>(“Intermediate” from [34]; 1/5 of “Strong”[d])	k_{19}=7.6e-2
**3B,0H**<br>*pThb10,12*	**140**<br>(“Weak” from [34])	k_{16}=5.2e-2 [e, f]
**1B,0H**<br>*pThb3*	**70**<br>(“Very Weak” from [34]; 1/3 of “Intermediate”[d])	k_{10}=3.6e-2

[a] RNA:protein ratio is set by the translation rate constant, k_{7A}=4e-1. The *hb* protein and mRNA decay constants (k_1 and k_{7B}, respectively) are 1.2e-1.
[b] Transcription rate set equal for 5 Bcd bound and 6 Bcd bound.
[c] pThb_ are the lacZ constructs from [34].
[d] Relative expression values from *in vitro* [32].
[e] Production for 2 Bcd bound was interpolated, k_{13}=4.4e-2.
[f] For the pThb15 (2 times 3B) and pThb16 (3 times 3B) constructs, k_{16} was raised to 1.8e-1, to match the stated “Strong” expression.
