# Supplementary material for: Gene Expression Noise in Spatial Patterning: hunchback Promoter Structure Affects Noise Amplitude and Distribution in Drosophila Segmentation
Source: PLoS Comput Biol. 2011 Feb 3;7(2):e1001069. doi: 10.1371/journal.pcbi.1001069 (PMC3033364; doi:10.1371/journal.pcbi.1001069)
Supplement: Table S2 — Bcd binding strengths. (0.04 MB PDF) [file pcbi.1001069.s014.pdf]

Table S2 – Bcd binding strengths

**Model** *Experiment*	**1A**[a] *pThb3*	**3A** *pThb10*	**3X** *pThb12*	**4A** *pThb11*	**4X** *pThb13*	**3A3X**[b] *hb^{14F}*	**2*x*(3X)**[c] *pThb15*	**3*x*(3X)**[d] *pThb16*
**Position of boundary** (%EL)	**35**	**35**	**21**	**42**	**23**	**42**	**28**	**30**
**Sharpness** (degrees)	**43**	**54**	**57**	**63**	**64**	**72**	**62**	**63**
**1st Bcd binding** (k_{11}, M^{-1} s^{-1})	2.8e7	2.8e7	1.09e7	2.8e7	1.09e7	2.8e7	1.09e7	1.09e7
**2nd Bcd** (k_{14})		3.6e7	1.67e7	3.6e7	1.67e7	3.6e7	1.67e7	1.67e7
**3rd Bcd** (k_{17})		4.18e7	1.94e7	4.18e7	1.94e7	4.18e7	3e8	4e8
**4th Bcd** (k_{20})				4.25e8	1.4e8	1.4e8		
**5th Bcd** (k_{23})						1.4e8		
**6th Bcd** (k_{26})						1.4e8		

These values are relative to unbinding rates (k_{12}, k_{15}, k_{18}, k_{21}, k_{24}, k_{27}) of 1/s. The model Bcd gradient was determined by k_8=3.5e-3 and k_9=2e-3, set to match the experimentally measured shape [3], and by a Bcd diffusivity D_{Bcd}=20μm^2/s (embryo-scale value measured in [57]).

[a] **A** strong site, **X** weak site (see Figure 2A).

[b] The core Bcd sites of the proximal promoter, no Hb self-binding.

[c] 2 copies of the 3X sequence; the 3rd site of the 3X segment was strengthened to match experimental position.

[d] 3 copies of the 3X sequence; the 3rd site of the 3X segment was strengthened to match experimental position.
