# Supplementary material for: Gene Expression Noise in Spatial Patterning: hunchback Promoter Structure Affects Noise Amplitude and Distribution in Drosophila Segmentation
Source: PLoS Comput Biol. 2011 Feb 3;7(2):e1001069. doi: 10.1371/journal.pcbi.1001069 (PMC3033364; doi:10.1371/journal.pcbi.1001069)
Supplement: Table S3 — Hb binding strengths. (0.04 MB PDF) [file pcbi.1001069.s015.pdf]

Table S3 – Hb binding strengths

**Model** *Experiment*	**3A3X, 2H** *WT*	**6B2H_lacZ**[b] *pThb5*	**6B1H_lacZ** *pThb1,6,9*	**2*x*(6B1H)_lacZ**[e] *pThb8*	**4B1H_lacZ**[f] *pThb2*
**Position of boundary** (%EL)	47	46	43	49	40
**Sharpness** (degrees**)**	83 *(protein)* 84[a] *(mRNA)*	78	71	75	52
**1st Hb bind** (k_3, M^{-1} s^{-1})	4e6	4e6	4e6[d]	4e6	4e6
**2nd Hb** (k_6)	1e8	1e8[c]			

Unbinding rates, k_4 and k_7, are set to 1/s.

[a] WT protein sharpness is fit to data, mRNA sharpness is a model prediction. Hb diffusivity D_{Hb}=0.3μm^2/s (equal to the nuclear-resolution value measured in [41]) gives the best fit to experimental sharpness.

[b] Expression levels for simulations with Hb sites were higher, but never more than twice, that of 6B0H (Table S1).

[c] The transcription rate (k_5) was dropped from 3.1 (WT) to 0.4, to match the loss of sharpness observed in this construct. Sharpnesses for the other constructs are model predictions.

[d] This value set by matching the posterior shift compared to *hb*^{14F}.

[e] 6th Bcd binding (k_{26}) set to 1e9, to match experimental posterior shift with construct doubling.

[f] This construct has a truncated *hb* promoter: 1st Bcd (A_1) binds at 2.8e7 (k_{11}); 2nd Bcd (A_2), 3.6e7 (k_{14}); 3rd Bcd (X_1), 2.5e7 (k_{17}; slightly stronger than in 4X, to match position); 4th Bcd (X_2), 1.4e8 (k_{20}; as in 4X and WT).
