# Supplementary material for: Gene Expression Noise in Spatial Patterning: hunchback Promoter Structure Affects Noise Amplitude and Distribution in Drosophila Segmentation
Source: PLoS Comput Biol. 2011 Feb 3;7(2):e1001069. doi: 10.1371/journal.pcbi.1001069 (PMC3033364; doi:10.1371/journal.pcbi.1001069)
Supplement: Text S1 — Deterministic modelling and experimental constraints set parameters. (0.05 MB PDF) [file pcbi.1001069.s016.pdf]

Text S1: Deterministic modelling and experimental constraints set parameters

The model was first solved deterministically - i.e., differential equations were solved for the time-evolution of the concentrations of the species in Figure 2B (plus Bcd and Hb diffusion) – to match large scale features of the data. These included maximum concentration, position of the Hb boundary (in percent egg length, %EL), and sharpness of the Hb boundary (degrees of inclination); using data from WT (protein and mRNA), the *hb*^{14F} mutant (protein), and the Driever et al. [34] reporter constructs (mRNA) with lacZ driven by portions of the *hb* promoter (in a WT background). 100 computational units (5 μm cubes) represented the AP axis, each unit corresponding to a nucleus and its associated cytoplasm (the 'energid' unit; see Text S2 on the processing of experimental data into energid units by Voronoi tessellation). As described in the Results, this allowed us to determine the binding constants, production and decay rates for mRNA and protein, and Hb diffusivity. All parameters are given in Tables S1 – S3.

Production rates (Table S1)

As described in the Results, we used the direct measurement of Bcd-GFP concentration [4] as an estimate of WT Hb concentration. Zamparo and Perkins [37] estimated several-fold lower values from fixed data, with techniques known to be biased low, but did estimate Bcd and Hb to be at similar concentrations. The Hb maximum in the model results from the sum of the transcription rates due to each of the 8 possible binding sites (Table S1, 3rd column). Concentrations in the mutant and constructs are estimated relative to WT (Table S1, 2nd column). We equated the "Strong" output found for lacZ constructs with 6 Bcd sites [34] with *hb*^{14F} expression. For the ratio between mRNA and protein, we estimated a fairly typical value of 1:35 [50] (mid-range for values in [36]). In the lacZ constructs, decreasing Bcd binding site number decreases expression intensity (Table S1, columns 1 and 2). Driever et al. rated these qualitatively [34], but quantitative expression ratios between some of the constructs are available from an earlier assay [32]. These were incorporated into the model, providing experimental constraints (Table S1, column 2) on transcription due to binding 1, 4 and 6 Bcds (Table S1, column 3); the transcription rate for 3 Bcds bound was interpolated from these (rates for 2 and 5 Bcds bound were also estimated, but have no corresponding data).

Bcd binding constants (Table S2)

The top row of Table S2 shows the expression boundary positions for the Driever lacZ constructs with no Hb binding sites (from [34]), and for the *hb*^{14F} mutant (from [30]). The different boundary positions for the different numbers of Bcd binding sites determine the binding strength at each of the sites; all experimental positions were reproduced by the model. For the 6 Bcd binding sites they identified in the proximal *hb* promoter, Driever and Nüsslein-Volhard [32] distinguished 3 as strong (A) and 3 as weak (X) binding sites, by DNAse footprinting (Figure 2A, red). The Driever et al. lacZ constructs cover many of the possible combinations of these A and X sites, allowing us to build up A and X binding strengths based on expression positions. Binding strengths are given relative to unbinding rates of 1/s (also see ref. [S1]).

The pThb3 construct has a single A site ("1A"), giving a starting point for the Bcd binding strengths. For the construct with 3 strong (A) sites (pThb10), we incorporated the relative increase in binding observed in vitro for the first three Bcd bindings [38], but found a smaller overall binding increase matched in vivo position. I.e. we retained the observed feature of a larger jump from 1st to 2nd binding than 2nd to 3rd binding, but in vivo positions suggest a smaller difference between 1st and 3rd binding strengths than measured in vitro (also see ref. [S2]

regarding in vivo cooperativity). Modelling the construct with 3 weak (X) sites (pThb12) suggests that weak sites have about 40% of the binding strength of strong sites, in vivo. The posterior shift observed in moving up to 4 sites (strong or weak) suggests a very strong cooperativity in binding the 4th Bcd: the model takes a roughly 10-fold increase in binding strength to match in vivo positions. For the *hb*^{14F} mutant, we modelled the 3A and 3X Bcd binding sites of the proximal *hb* promoter (Figure 2A, red), with no Hb binding. We found that the 5th and 6th Bcds can bind at equal strength as the 4th Bcd; the observed posterior shifts can result simply from increasing the number of Bcd sites, rather than stronger binding. Two more constructs directly address binding site number: pThb15 has a doubling of the 3X sequence, and pThb16 has a tripling of 3X. In these cases, we modelled the doubling and tripling of the binding sites, respectively, leaving the first two binding strengths unchanged. However, matching the posterior shifts of these constructs required increases in the 3rd binding strength, suggesting increased cooperativity upon multiplication of the 3X sequence (c.f. the 3rd to 4th Bcd jump above).

Hb regulation (Table S3)

The addition of the 2 Hb sites (Figure 2A, blue, within the green arrows), at the strengths shown (Table S3, column 1), produces WT position and sharpness. Cooperative loading of the promoter, with the 2nd Hb binding much more strongly than the 1st, produces a stationary boundary; the relative strengths for the 2 Hb bindings are derived from the posterior shifts and sharpening from *hb*^{14F} to pThb1 to pThb5 to WT. The high transcription rate once the 2nd Hb is bound (Table S1) produces the observed large expression jump from *hb*^{14F} to WT. The model predicts slightly sharper mRNA than protein, as was verified experimentally [30, Table 1]. Model sharpness was matched to WT protein, giving somewhat higher than experimental values for *hb*^{14F} (72° vs. 64°) and pThb5 (78° vs. 73°). All other sharpnesses in Tables S2 and S3 (2nd rows) are predictions relative to these benchmarks, which could be tested experimentally.

Setting the Hb production and decay rates to match the observed steady sharpness and maximum at 30 minutes (Table S1) indicates a fairly short half-life for the Hb protein, of about 1 minute. For the dynamics considered, reaching the same maximum with a much longer half-life would require a too-slow accumulation of protein, such that the pattern would not become steady within the developmental timeframe. Fast equilibration of mRNA, with a short mRNA half-life (expression data suggests mRNA is more transient than protein (ref. [S3]); http://bdtnp.lbl.gov/Fly-Net/), does not alter the constraints on the protein constants.

Supplementary References

S1. Krishna S, Banerjee B, Ramakrishnan TV, Shivashankar GV (2005) Stochastic simulations of the origins and implications of long-tailed distributions in gene expression. Proc Nat Acad Sci USA 102: 4771 – 4776.

S2. Lebrecht D, Foehr M, Smith E, Lopes FJP, Vanario-Alonso CE, et al. (2005) Bicoid cooperative DNA binding is critical for embryonic patterning in *Drosophila*. Proc Nat Acad Sci USA 102: 13176-13181.

S3. Fowlkes CC, Luengo Hendriks CL, Keränen SVE, Weber GH, Rübel O, et al. (2008) A quantitative spatio-temporal atlas of gene expression in the *Drosophila* blastoderm. Cell 133: 364-374.
