# Supplementary material for: Gene Expression Noise in Spatial Patterning: hunchback Promoter Structure Affects Noise Amplitude and Distribution in Drosophila Segmentation
Source: PLoS Comput Biol. 2011 Feb 3;7(2):e1001069. doi: 10.1371/journal.pcbi.1001069 (PMC3033364; doi:10.1371/journal.pcbi.1001069)
Supplement: Text S2 — Image processing and analysis. (0.02 MB PDF) [file pcbi.1001069.s017.pdf]

Text S2. Image Processing and Analysis

The image processing routines involve three main stages: (i) nuclear identification; (ii) energid identification; and (iii) averaging expression intensity.

(i) Nuclear identification: a fully automatic segmentation routine was developed in order to identify the nuclei of the embryos. This routine consists of successive thresholds and connected component labeling of the nuclei in order to avoiding merging between neighbouring nuclei [65]. The result of the segmentation is a nuclear mask image (e.g. Figure S1). Protein expression, rather than mRNA data, was used to identify nuclei; the higher protein intensity more clearly shows the morphology of the nuclei.

(ii) Energid identification: after nuclear detection, we proceeded to identify the cytoplasmic region associated with each nucleus (each nucleus with its associated cytoplasm is an energid unit). This was accomplished by using the generalized Voronoi diagrams of the nuclear mask image [66]. In this process, the nuclei of the nuclear mask image were labeled and each of the remaining pixels of the image received the label of its closest nucleus. Figure S2 shows the generalized Voronoi diagram of the image in Figure S1.

(iii) Averaging expression intensity: the average intensity of the pixels inside each energid was used to quantify the mRNA or protein expression levels. Protein data was first used to identify the energids, but then average intensity was computed for both protein (e.g. Figure S3A) and mRNA data (e.g. Figures S3B,C). This data was used for comparison to simulations.
