# Supplementary material for: Gene Expression Noise in Spatial Patterning: hunchback Promoter Structure Affects Noise Amplitude and Distribution in Drosophila Segmentation
Source: PLoS Comput Biol. 2011 Feb 3;7(2):e1001069. doi: 10.1371/journal.pcbi.1001069 (PMC3033364; doi:10.1371/journal.pcbi.1001069)
Supplement: Text S3 — Trend extraction with 2D-SSA. (0.03 MB PDF) [file pcbi.1001069.s018.pdf]

Text S3. Trend extraction with 2D-SSA

1 Trend extraction process

The aim of this procedure is to decompose the expression data values into a sum of “trend” + “noise”, where “trend” is a smooth general 2D expression pattern. We assume that the data is given at nuclear centres (for mRNA data an additional preprocessing step is needed). Smoothing algorithms are generally designed for data defined on regular grids (e.g. a regularly-spaced rectangular grid). Because of this, we perform smoothing in three steps:

1. Regularization of data.
2. Smoothing with 2D-SSA.
3. Reconstruction of values at nuclear centres.

All steps of the procedure were implemented as a Matlab script; the 2D-SSA smoothing was performed with the 2D-SSA application [72].The analysis shown in Figures S4-S12 are for the same embryo and data as Figure 3C of the main text.

1.1 Regularization

In the first step, the expression data is converted into a function defined on a regular grid. An appropriate rectangular cropping area is selected (see Figure S4). It should have no blank parts. Next, this data is interpolated on a regular grid (Figure S5).

A linear interpolation is used (the function is approximated by a piecewise linear function, defined on triangles given by a Delaunay triangulation). The resolution of the regular grid should be small in comparison to inter-nuclear distances (0.2%EL was used for all analyses). Figure S6 shows a greyscale plot of the regularized data.

1.2 Smoothing

Data smoothing on the regular grid is performed with the 2D-SSA procedure; further details on this method can be found in [70,71]. In brief, 2D-SSA consists of construction of a so-called trajectory matrix by means of sliding 2D windows of given sizes; decomposition of this matrix into a set of elementary matrices ordered by their contribution to the decomposition; and then reconstruction of 2D patterns based on the chosen set of elementary decomposition components. The choice of parameters is discussed in subsection 1.3. 2D-SSA produces a smooth function defined on the same regular grid (see Figure S7).

1.2.1 Reconstruction of values at nuclear centres

To determine the values on the original irregular grid (points of nuclear centres) a bilinear interpolation is used; the values are interpolated separately along X and Y. (For an appropriately small regularization step, the particular interpolation method is not significant.) The noise (the residuals) is calculated by subtracting the trend from the original data for each energid.

1.3 Choice of parameters in 2D-SSA

Smoothing by 2D-SSA, one has to select two parameters: (1) the size of the sliding 2D window, and (2) the number of components in the SSA decomposition corresponding to the trend. If the pattern has a smooth but non-regular form, then the window sizes should be small relative to the regularized image size. With this choice of parameters, the trend is extracted by 2D-SSA on the basis of a small number of leading components of the SSA decomposition. Since the set of the chosen (trend) components should correlate weakly with the residual (noise) set, one can determine the number of trend components by examining elementary components and

the plot of w-correlations between components. In Figure S8, the first two or three components correspond to the trend, forming a block which is weakly correlated with the rest.

Window sizes are also significant for the smoothing resolution. They must be large enough to capture enough nuclei. Smaller windows give more details in the leading components (i.e., the pattern is captured by just one or two leading components), while larger windows give better resolution and decompose the trend into a larger set of components. In Figure S9 and Figure S10, the initial expression data in a specified strip is shown with the 2D-SSA trend (taken from the regularized grid line in the middle of the strip). Data is plotted for different window sizes. The 33x33 window gives a sufficiently smooth and accurate pattern.

Choosing the number of components can also affect the fit. Figure S10 shows the 33x33 window fit with 2 components; it can be seen that this has a worse fit at low data values than does the fit with 3 trend components shown in Figure S9. The effect of component number on fit can also be seen in the residual plots of Figure S11.

From these considerations, we have chosen a window size of 33x33 and 3 components in this study. Since the expression patterns in this work are similar, we use these parameters for all embryos, for comparison between data sets.

1.4 Dependence of noise on trend

Figure S12 plots the dependence of residuals on trend values for the same embryo (left, absolute residuals; right, relative residuals (absolute/trend)). Moving averages are marked black and moving standard deviations are marked red. Both statistics are calculated over 30 adjacent trend values. Noise (residuals) is dependent on intensity (trend), i.e. it shows a multiplicative character.
